# Identification of cardioprotective drugs by medium-scale *in vivo* pharmacological screening on a *Drosophila* cardiac model of Friedreich's ataxia

**DOI:** 10.1242/dmm.033811

**Published:** 2018-07-20

**Authors:** Amandine Palandri, Elodie Martin, Maria Russi, Michael Rera, Hervé Tricoire, Véronique Monnier

**Affiliations:** Université Paris Diderot, Sorbonne Paris Cité, Unité de Biologie Fonctionnelle et Adaptative (BFA) UMR8251 CNRS, 75205, Paris Cedex 13, France

**Keywords:** *Drosophila*, Cardiomyopathy, Pharmacological screening, Friedreich’s ataxia, Microtubule, Methylene Blue

## Abstract

Friedreich's ataxia (FA) is caused by reduced levels of frataxin, a highly conserved mitochondrial protein. There is currently no effective treatment for this disease, which is characterized by progressive neurodegeneration and cardiomyopathy, the latter being the most common cause of death in patients. We previously developed a *Drosophila melanogaster* cardiac model of FA, in which the fly frataxin is inactivated specifically in the heart, leading to heart dilatation and impaired systolic function. Methylene Blue (MB) was highly efficient to prevent these cardiac dysfunctions. Here, we used this model to screen *in vivo* the Prestwick Chemical Library, comprising 1280 compounds. Eleven drugs significantly reduced the cardiac dilatation, some of which may possibly lead to therapeutic applications in the future. The one with the strongest protective effects was paclitaxel, a microtubule-stabilizing drug. In parallel, we characterized the histological defects induced by frataxin deficiency in cardiomyocytes and observed strong sarcomere alterations with loss of striation of actin fibers, along with full disruption of the microtubule network. Paclitaxel and MB both improved these structural defects. Therefore, we propose that frataxin inactivation induces cardiac dysfunction through impaired sarcomere assembly or renewal due to microtubule destabilization, without excluding additional mechanisms. This study is the first drug screening of this extent performed *in vivo* on a *Drosophila* model of cardiac disease. Thus, it also brings the proof of concept that cardiac functional imaging in adult *Drosophila* flies is usable for medium-scale *in vivo* pharmacological screening, with potent identification of cardioprotective drugs in various contexts of cardiac diseases.

## Abstract (precis)

**Editor's choice:** The first medium-scale pharmacological *in vivo* screen on a *Drosophila* cardiac model identified 11 compounds with protective effects on Friedreich's ataxia cardiomyopathy.

## INTRODUCTION

Friedreich's ataxia (FA) is the most frequent autosomal recessive spinocerebellar ataxia in Caucasians, with a prevalence of around 1 in 50,000. The first symptoms usually appear around puberty and most cases develop before the age of 25 years, with a life expectancy of approximately 37 years (Delatycki et al., 2000). The main feature of FA disease is the progressive ataxia, with patients losing the ability to walk usually 10 to 15 years after the onset of the disease. Other neurological features include dysarthria, tendon areflexia, sensory loss and pyramidal signs (Dürr et al., 1996; Harding, 1981; Pandolfo, 2009; Tsou et al., 2011). Besides the neurological involvement, FA patients also present a cardiomyopathy that is the leading cause of death (Casazza and Morpurgo, 1996; Child et al., 1986; Dürr et al., 1996; Dutka et al., 2000; Giunta et al., 1988; Hawley and Gottdiener, 1986; Kipps et al., 2009; Morvan et al., 1992; Unverferth et al., 1987; Weidemann et al., 2012). The cardiomyopathy consists of left ventricular (LV) hypertrophy that may develop into dilated cardiomyopathy and systolic dysfunction in end-stage hearts (Regner et al., 2012). At the histological level, cardiomyocyte hypertrophy, diffuse fibrosis and focal myocardial necrosis have been reported (Koeppen, 2011; Raman et al., 2011; Unverferth et al., 1987).

FA is caused by a GAA trinucleotide repeat expansion in the first intron of *FXN*, the gene encoding frataxin. This results in a decreased gene expression and partial loss of function of the protein (Campuzano et al., 1996). Frataxin is a highly conserved mitochondrial protein that regulates the early steps of biogenesis of iron-sulfur clusters (Fe-S), which are essential protein cofactors involved in a large number of cellular functions (Braymer and Lill, 2017). Frataxin deficiency leads to several cellular dysfunctions, including decreases in aconitase and mitochondrial respiratory chain activities (Rötig et al., 1997), hypersensitivity to oxidative stress (Wong et al., 1999) and accumulation of intra-mitochondrial iron that is associated with depletion of cytosolic iron in affected organs (Babcock et al., 1997; Huang et al., 2009; Puccio et al., 2001). Several pharmacological compounds have been or are currently being evaluated in FA patients, including antioxidants (e.g. idebenone), iron chelators (e.g. deferiprone) or compounds that could increase frataxin protein levels (e.g. histone deacetylase inhibitors or erythropoeitin) (reviewed in Aranca et al., 2016). However, there are no existing pharmacological strategies leading to sustained clinical improvement. It is therefore necessary to better understand the pathophysiological mechanisms involved in this complex disease and to identify new candidate molecules for pharmacological treatments.

Several unbiased pharmacological screens have already been performed on frataxin-deficient cells. A screen on murine fibroblasts with partial frataxin deficiency, using a targeted ribozyme strategy, failed to identify potent hits and was limited by instability of the cellular model (Calmels et al., 2009). Two screens were also performed on yeast cells, using a marker of mitochondrial function or rescue of defective growth as readouts, and led to the identification of several active compounds (Cotticelli et al., 2012; Seguin et al., 2015). Besides these screening approaches based on cellular models, the use of animal models for *in vivo* pharmacological screens appears particularly relevant in that they allow the identification and evaluation of new compounds at the level of the whole organism or more specifically on the function of specific organs. In particular, given the importance of cardiac involvement in FA, the identification of compounds capable of improving the function of frataxin-deficient hearts would be of particular interest. The mouse MCK model of FA, a conditional mouse model with complete frataxin deletion in cardiac and skeletal muscle, has already allowed evaluation of candidate compounds, such as idebenone and iron chelators (Seznec et al., 2004; Whitnall et al., 2008). However, mouse models are not suitable to screen and evaluate *in vivo* a large number of compounds. In contrast, such approaches are conceivable using *Drosophila* models. Indeed, several models of FA have already been generated in *Drosophila melanogaster*, mainly based on RNAi-mediated downregulation of *fh*, the gene encoding the fly frataxin homolog, ubiquitously or in specific tissues (Anderson et al., 2005, 2008; Llorens et al., 2007; Navarro et al., 2010). Using this strategy, we have previously generated a *Drosophila* cardiac model of FA in which *fh* was inactivated specifically in the fly heart. This model presents heart dilatation and impaired systolic function that are fully rescued by complementation with human frataxin (Tricoire et al., 2014). Thus, this model recapitulates defects of cardiac function observed in patients and mouse models of FA (Puccio et al., 2001; Seznec et al., 2004; Weidemann et al., 2012), and highlights conserved cardiac functions of frataxin between *Drosophila* and mammals. It already allowed the identification of Methylene Blue (MB) as a highly protective compound to prevent cardiac dysfunction and it was used to evaluate compounds preselected in a yeast screen (Seguin et al., 2015; Tricoire et al., 2014). The cardiac imaging method that we developed in flies allows studies on large populations: movie acquisition is fast and all the steps of the analysis are automated (Monnier et al., 2012). Thus, it seemed appropriate to conduct a medium-scale pharmacological screen on this model. In this study, we have screened *in vivo* the Prestwick Chemical Library, a library composed of 1280 approved drugs with known bioavailability and safety in humans, in search of compounds preventing dilatation of frataxin-deficient hearts. We also characterized the structural defects induced by frataxin deficiency in cardiomyocytes and tested the effects of two lead compounds, paclitaxel (identified in the screen) and MB, on these cellular phenotypes.

## RESULTS

### Primary screening of the Prestwick Chemical Library and drug validation

**Fig. 1.**
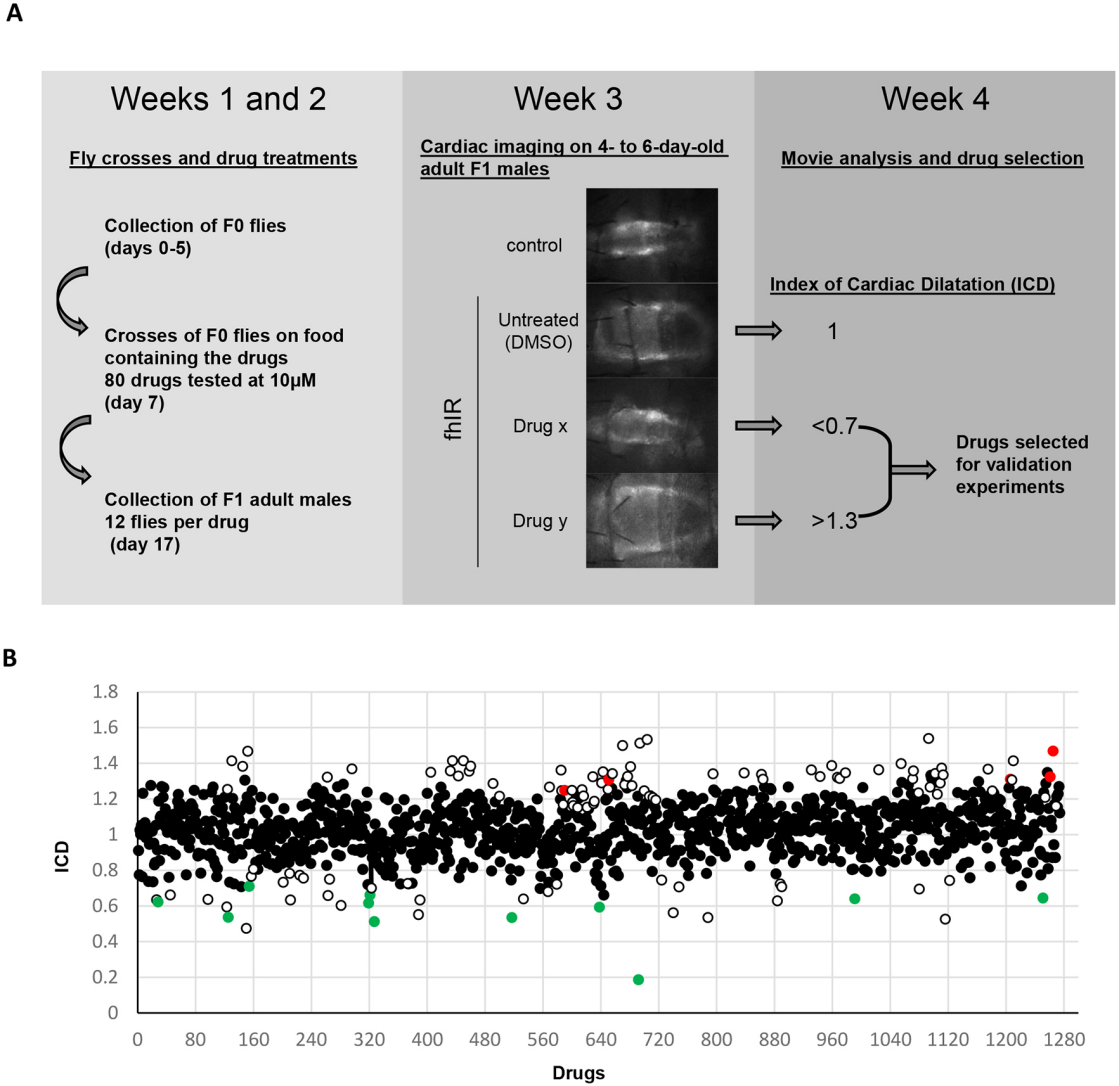
**Primary screen of** **the Prestwick Chemical Library.** (A) Scheme of the screening procedure. F0 flies were *UAS-fhIR* males and *UAS-mitoGFP; Hand-GS* females. F1 male flies were *UAS-mitoGFP*/*UAS-fhIR; Hand-GS*/*+* (named fhIR flies). F1 individuals developed on medium containing RU486, to induce frataxin inactivation, and the drugs to be tested. F1 adult males were collected at emergence, transferred to a new medium containing RU486 (without the drugs) and submitted to cardiac imaging at 4 to 6 days of age. (B) Index of cardiac dilatation (ICD) obtained during the primary screen for the 1275 tested drugs. White circles correspond to drug selected during the primary screen. Green and red circles correspond to validated drugs with protective or aggravating effects, respectively.

We tested the drugs *in vivo* on *UAS-mitoGFP*/*UAS-fhIR; Hand-GS*/*+* flies (named hereafter fhIR flies), in which the *Drosophila* frataxin is downregulated by RNA interference (RNAi) under control of the heart-specific and RU486 (mifepristone)-inducible *Hand-GS* driver. This driver also controls the expression of a mitochondrial GFP (mitoGFP), providing sufficient fluorescence in cardiomyocytes for high-speed video recording through the cuticle of anesthetized flies (Monnier et al., 2012). The primary screening was organized in 16 successive experiment subsets. Each subset allowed the testing of 80 compounds and required 4 weeks of experimental work including collection and crosses of F0 flies, development of the progeny on media containing the compounds at 10 µM, selection and collection of F1 adult fhIR male flies, cardiac imaging, and movie analysis (Fig. 1A). Since cardiac imaging was performed on 4- to 6-day-old flies, we first checked that the cardiac phenotype was stable over this age window in untreated fhIR controls (Fig. S1). In each experiment, cardiac imaging was performed on 12 fhIR flies per drug, along with 70 fhIR and 20 wild-type control (*UAS-mitoGFP*/*+; Hand-GS*/*+*) untreated flies, leading to a total of more than 16,000 individuals analyzed for cardiac function during the whole primary screen.

Only five compounds could not be evaluated for their effect on heart function, owing to their toxicity. These are three compounds with known insecticidal properties (ivermectin, avermectin 1A and trichlorfon) as well as two compounds used in oncology for their antineoplastic properties (camptothecin and gemcitabine). For the 1275 other compounds, we estimated the diastolic diameter (DD) and calculated an index of cardiac dilatation (ICD; see Materials and Methods for formulas). By definition, within each experiment subset, the value of this index is 1 in untreated fhIR flies and 0 in control flies. Compounds were selected sequentially in each experiment subset when the ICD was lower than 0.7 or higher than 1.3 with a *P*-value (obtained by comparing the DDs of treated and untreated fhIR flies) below 5×10^−2^. Compounds with a *Z*-score >2, calculated at the end of the primary screen on the full data, were also selected. With these criteria, we selected 43 compounds improving the heart dilatation, and 80 compounds worsening it (Fig. 1B). Validation experiments were then performed on larger samples per drug. We used at this step a movie analysis procedure allowing the extraction of end-diastolic and end-systolic diameters (EDD and ESD, respectively), and consequently the calculation of indexes of diastolic and systolic dilatation (IDD and ISD, respectively). Similarly to ICD, the values of IDD and ISD are by definition equal to 1 in untreated fhIR flies, and 0 in controls. The validation experiments allowed us to restrict the list of statistically significant compounds to 11 suppressors and five enhancers (presented in Table 1).

**Table 1.**
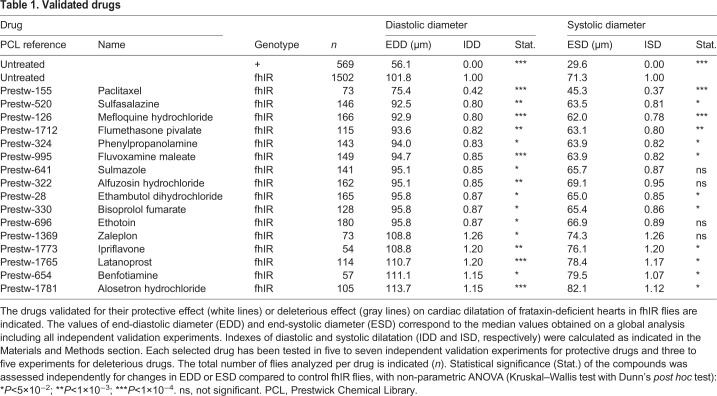
**Validated drugs**

The compound with the strongest protective effect on cardiac dilatation was paclitaxel, which had an IDD of 0.42. This drug, also known as Taxol, is a microtubule (MT)-stabilizing drug. In fhIR untreated flies, EDD was 81% higher than in control flies, and decreased to only 34% following treatment with paclitaxel in fhIR flies. The other ten compounds improved cardiac dilatation more modestly but significantly, with IDDs between 0.8 and 0.87. We also compared the systolic diameters of treated and untreated fhIR flies. The ESD of untreated fhIR flies was 141% higher than in control flies. ESDs were significantly decreased by treatment with eight of the 11 drugs selected for their effect on the DD. Here again, paclitaxel was the more efficient compound, with an ESD only 53% higher than in controls, leading to an ISD of 0.37. We also tested the protective compounds at other concentrations (Table S1). Although 30 μM treatments did not further improve the effects on cardiac dilatation compared to 10 µM treatments, we observed stronger effects with 1 µM treatments for two drugs, sulmazole and ethambutol (Fig. S2). This suggests the existence of complex dose-response effects in this context of *in vivo* screening in flies. The nature of the selected compounds, their pharmacological properties and possible relevance in the context of FA are discussed in the following section.

We have also identified five drugs that worsened cardiac dilatation in fhIR flies. These drugs were specific to frataxin deficiency, since they did not induce heart dilatation nor affected contractility of wild-type flies (Fig. S3).

### Paclitaxel prevents heart dilatation and improves contractility of frataxin-deficient hearts in a dose-dependent manner

**Fig. 2.**
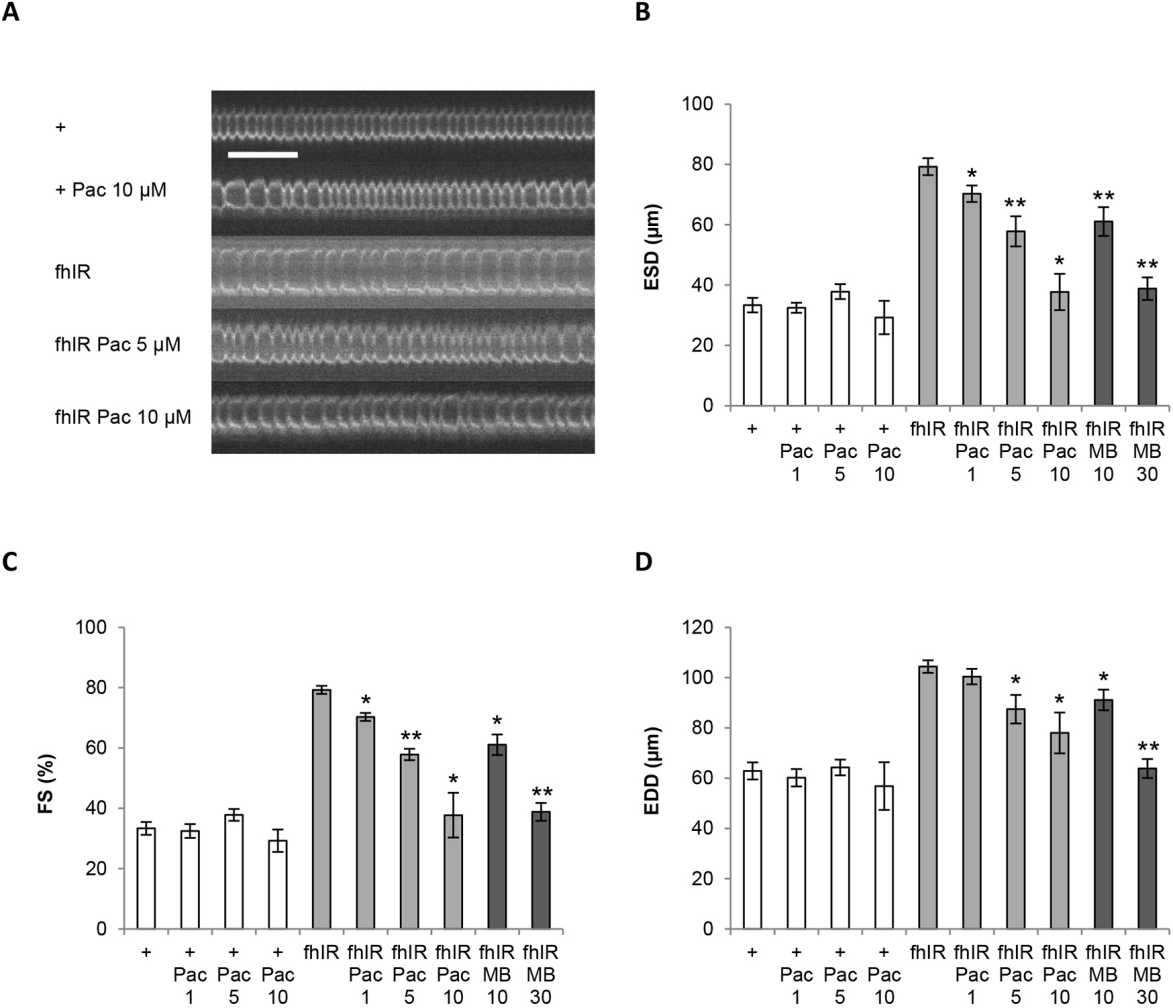
**Dose-dependent effect of paclitaxel treatment on cardiac function of frataxin-deficient hearts.** (A) Representative M-modes (generated by horizontal alignment of rows extracted at the same position for each movie frame) of control *UAS-mitoGFP*/*+; Hand-GS*/*+* (+) flies, untreated or treated with 10 µM paclitaxel, and of *UAS-mitoGFP*/*UAS-fhIR; Hand-GS*/*+* (fhIR) flies, untreated or treated with 5 µM or 10 µM paclitaxel. Scale bar: 1 s. (B-D) End-systolic diameter (ESD, µm), fractional shortening (FS, %) and end-diastolic diameter (EDD, µm) of + control flies, treated with DMSO (*n*=17), or with 1 µM (*n*=15), 5 µM (*n*=10) or 10 µM paclitaxel (*n*=5), and of fhIR flies treated with DMSO (*n*=19), or with 1 µM (*n*=22), 5 µM (*n*=15), 10 µM paclitaxel (*n*=10), or with 10 µM (*n*=18) or 30 µM Methylene Blue (MB; *n*=20). All flies were 4 days old and fed with RU486 during both development (40 ng/ml of food) and adulthood (100 µg/ml). All values are means (±s.e.m.). Statistical significance was assessed by non-parametric Wilcoxon analysis. Significant differences between treated and untreated flies of the same genotype are indicated: *P<5×10^−2^; **P<1×10^−3^.

We then focused our study on the lead compound, paclitaxel. To exclude artifacts, we first checked that paclitaxel did not modify the RNAi-mediated decrease of *fh* mRNA level (Fig. S4). Then, we performed dose-response assays. We could not test paclitaxel at concentrations higher than 10 µM, since it affected the development of flies, with partial pupal lethality. So, we treated flies with increasing doses from 1 to 10 µM (Fig. 2). In this concentration range, paclitaxel treatment did not affect heart function of control flies. We observed a dose-dependent effect of paclitaxel treatment on ESD, EDD and fractional shortening (FS) of fhIR flies. Noticeably, in this experiment, ESD of untreated fhIR flies was 138% higher than in control flies, whereas they were only 111%, 73% and 13% higher, respectively, in fhIR flies treated with 1 µM, 5 µM and 10 µM of paclitaxel (Fig. 2B). This compound was also particularly efficient in improving heart contractility, with a FS similar to wild-type flies following a 10 µM treatment (Fig. 2C). EDD was also progressively improved, although to a lesser extent: EDD of untreated fhIR flies was 66% higher than controls, and only 60%, 39% and 24% higher, respectively, following treatment with 1 µM, 5 µM and 10 µM of paclitaxel. Representative movies of a control fly heart (Movie 1), and fhIR hearts untreated (Movie 2) or treated with 10 µM paclitaxel (Movie 3) are also provided. MB was used in this experiment as a positive control at 10 µM and 30 µM. Consistent with our previous study (Tricoire et al., 2014), this latter concentration fully prevented cardiac dilatation of frataxin-deficient hearts (Fig. 2B-D). However, at similar molar concentrations (10 µM), paclitaxel was more efficient than MB in improving ESD and FS. It should be noted that both ESD and EDD of fhIR hearts treated with 10 µM paclitaxel were statistically different from ESD and EDD of wild-type control hearts when statistical analysis was performed on the full set of data presented in Table 1 (*P*<1×10^−4^), showing that paclitaxel treatment only led to a partial rescue at this concentration. We could not determine whether higher concentrations of paclitaxel would be able to improve DD as efficiently as MB at 30 μM, due to the narrow therapeutic window of this compound. We also studied the effects of paclitaxel post-symptomatic treatment. For this, we treated fhIR flies with paclitaxel only at the adult stage and performed cardiac imaging on 10-day-old flies. This post-symptomatic treatment did not improve cardiac function (Fig. S5).

### Histological characterization of frataxin-deficient hearts

**Fig. 3.**
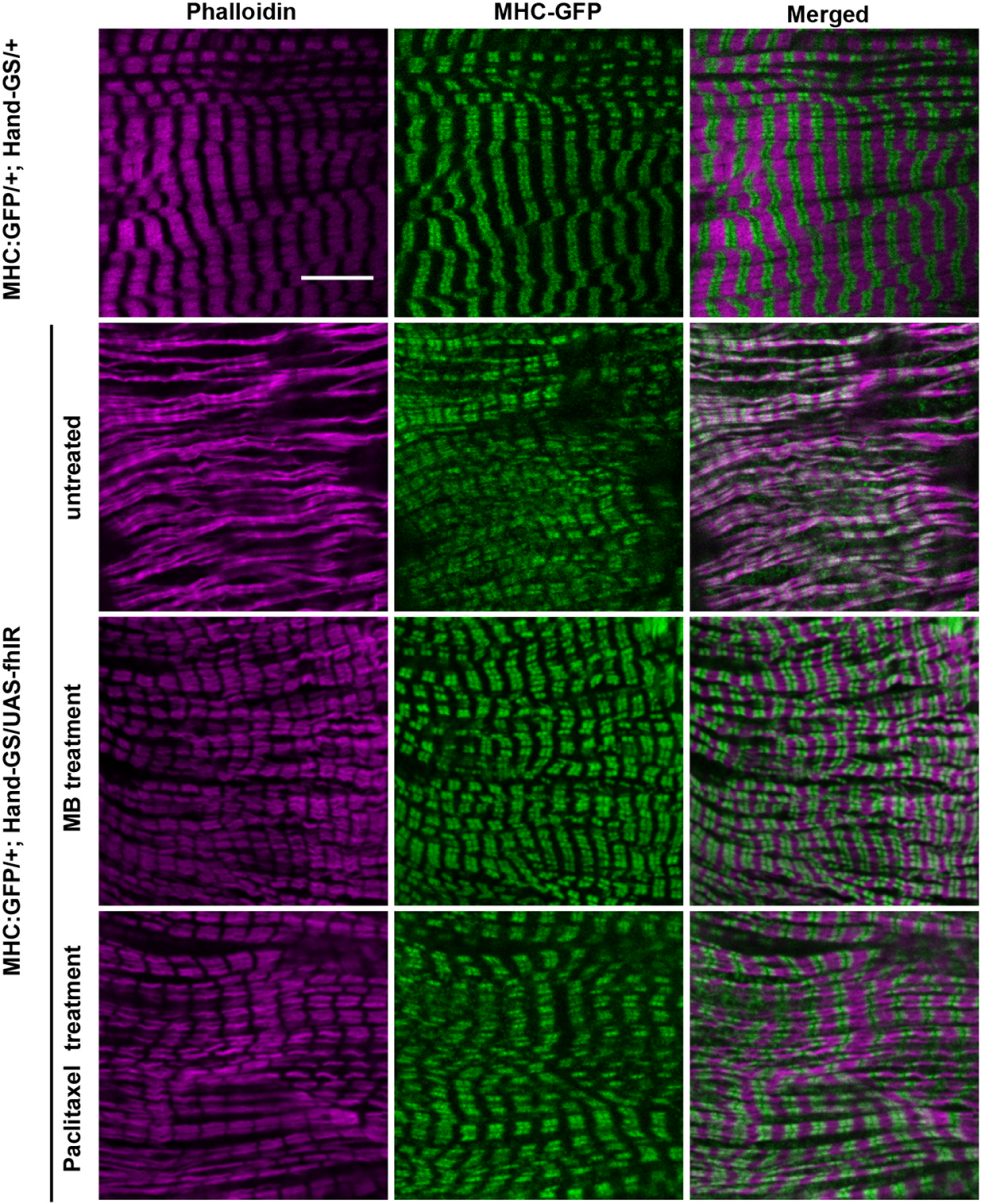
**The actin and myosin networks are affected by frataxin depletion in cardiomyocytes and improved by paclitaxel and MB treatments.** Hearts of 3- to 5-day-old adult male flies were dissected and double-labeled with phalloidin to stain F-actin and an anti-GFP antibody to stain the MHC-GFP fusion protein. *MHC:GFP*/*+; Hand-GS*/*+* control flies were treated with DMSO (i.e. untreated; top row). *MHC:GFP*/*UAS-fhIR; Hand-GS*/*+* flies were treated with DMSO (untreated), 30 µM MB or 10 µM paclitaxel. All flies were fed with RU486 during both development (40 ng/ml of food) and adulthood (100 µg/ml). Scale bar: 10 µm.

**Fig. 4.**
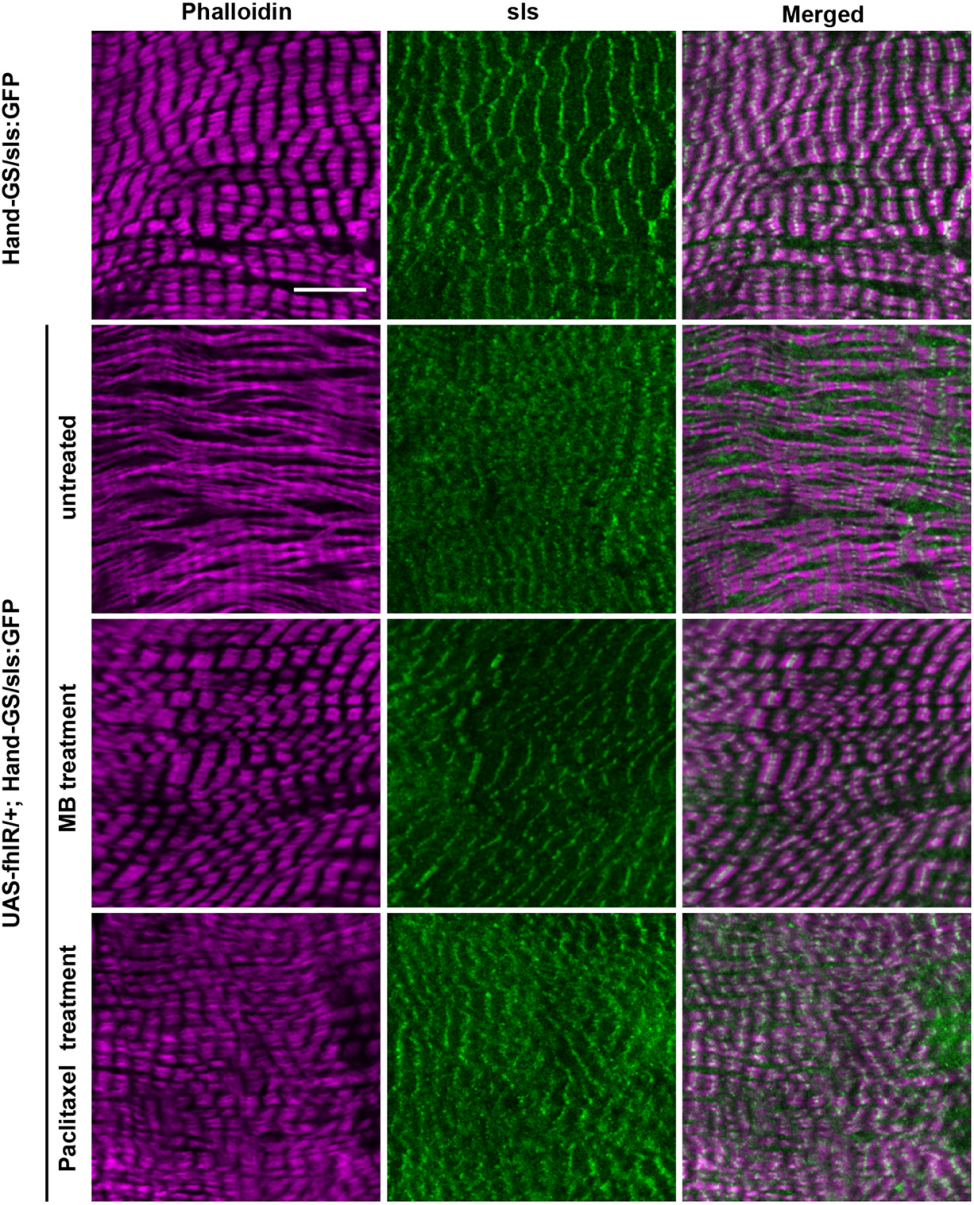
**The sarcomeric pattern of Sallimus is modified in frataxin-depleted hearts, and improved by paclitaxel and MB treatments.** Hearts of 3- to 5-day-old adult male flies were dissected and double-labeled with phalloidin to stain F-actin and an anti-GFP antibody to stain the Sls-GFP fusion protein. *Hand-GS*/*sls:GFP* control flies were treated with DMSO (i.e. untreated; top row). *UAS-fhIR*/*+; Hand-GS*/*sls:GFP* flies were treated with DMSO (untreated), 30 µM MB or 10 µM paclitaxel. All flies were fed with RU486 during both development (40 ng/ml of food) and adulthood (100 µg/ml). Scale bar: 10 µm.

**Fig. 5.**
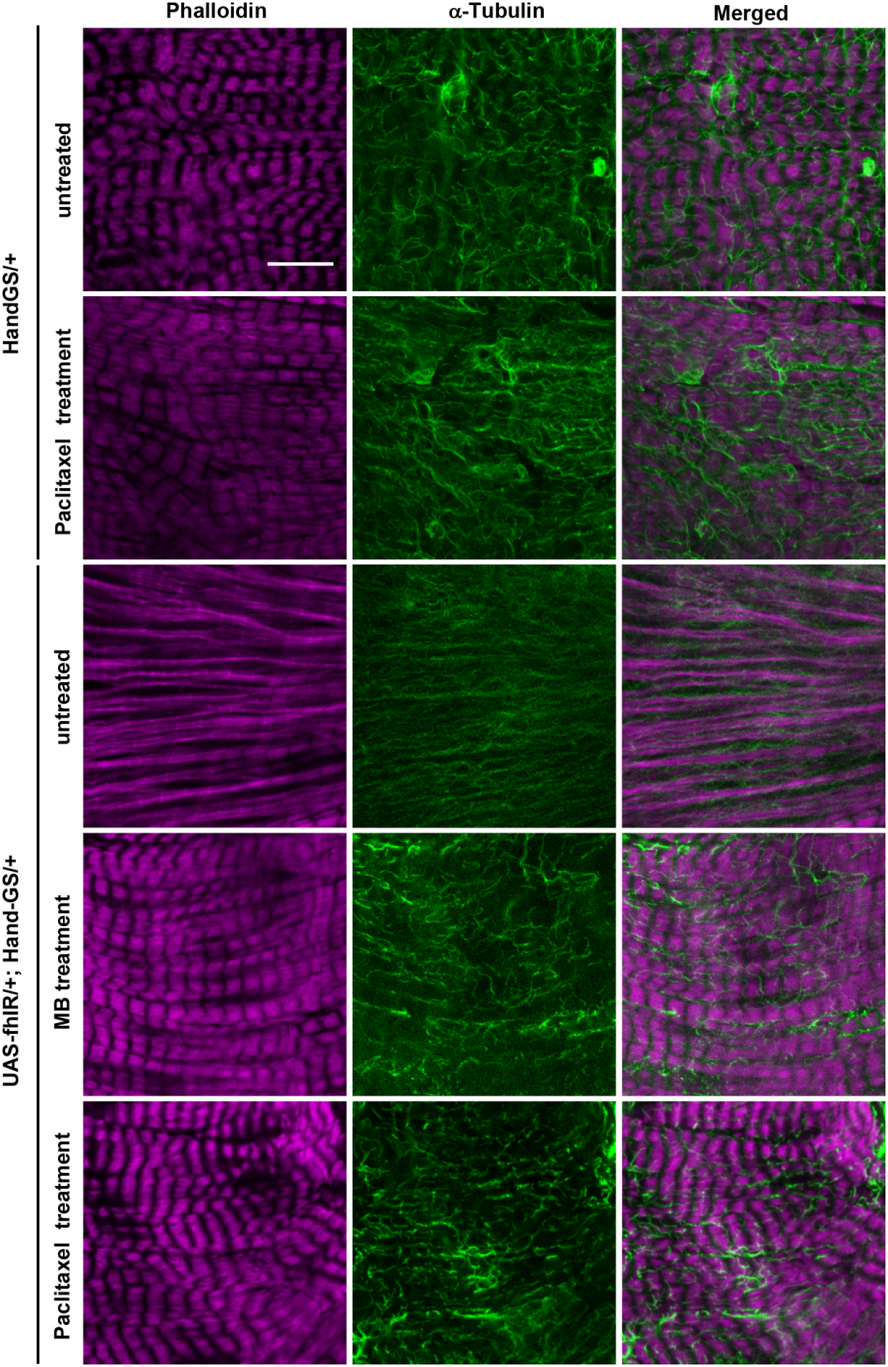
**Disruption of the microtubule network in frataxin-depleted hearts is prevented by paclitaxel and MB treatments.** Hearts of 7-day-old adult male flies were dissected and double-labeled with phalloidin to stain F-actin and an anti-α-tubulin antibody. *Hand-GS*/*+* control flies were treated with DMSO (untreated) or with 10 µM paclitaxel. *UAS-fhIR*/*+; Hand-GS*/*+* flies were treated with DMSO (untreated), 30 µM MB or 10 µM paclitaxel. All flies were fed with RU486 during both development (40 ng/ml of food) and adulthood (100 µg/ml). Scale bar: 10 µm.

Next, we characterized the structural defects induced by frataxin deficiency in cardiomyocytes, in order to better understand the causes of cardiac functional defects and to ultimately study the effects of compounds identified by functional pharmacological screening on these structural phenotypes. To this purpose, we used two protein trap lines with GFP exons inserted in genes encoding two sarcomeric proteins: the *Myosin heavy chain* gene (*MHC*) and *Sallimus* (*Sls*) (Morin et al., 2001; Orfanos et al., 2015). We first characterized sarcomeric organization in cardiomyocytes of *MHC:GFP*/*UAS-fhIR; Hand-GS*/*+* flies compared to *MHC:GFP*/*+; Hand-GS*/*+* control flies. Phalloidin and GFP immunostaining were used to observe the F-actin and myosin networks, respectively (Fig. 3). The periodic F-actin striations, observed in control flies, were fully absent in cardiomyocytes of frataxin-depleted hearts. Myosin striations associated with actin fibers were partly present, but highly irregular. Diffuse and punctuate MHC-GFP staining, unassociated with F-actin, was also observed. Then, Sls distribution was observed in *UAS-fhIR*/*+; Hand-GS*/*sls:GFP* flies and compared to *Hand-GS*/*sls:GFP* control flies. Sls is a large protein similar to the I-band region of the vertebrate titin, with various isoforms expressed in different muscle types and linking the Z-disc to the A-band (Burkart et al., 2007). The Sls-GFP fusion protein was previously shown to display a Z-disc pattern in indirect flight muscles of *Drosophila* (Orfanos et al., 2015). Here, we observed a similar Z-disc pattern in cardiomyocytes of control flies (Fig. 4). In frataxin-depleted hearts, although a periodic pattern was distinguishable, the GFP staining was more diffuse and irregular. Finally, considering the cardioprotective effect of paclitaxel shown above, we also characterized the MT network. In control *Hand-GS*/*+* flies, α-tubulin immunostaining revealed a dense network of MT interspersed between the myofibrils, whereas, in *UAS-fhIR*/*+; Hand-GS*/*+*flies, the MT network was fully disrupted (Fig. 5). Therefore, frataxin deficiency during development induced striking disorganization of the cardiomyocyte sarcomeres and of their associated MT network. The sarcomeric organization and MT network of longitudinal muscle fibers spreading along the ventral side of the heart, in which the *Hand-GS* driver is not expressed, were not affected in fhIR flies, showing that these structural defects were cell-autonomous (Fig. S6). Interestingly, the actin network was progressively restored following arrest of RU486-induced frataxin inactivation in adult fhIR flies (Fig. S7). On the other hand, inactivation of frataxin only during the adult stage did not induce sarcomere disorganization or cardiac dilatation, nor affect the longevity (Fig. S8). It should be noted that developmental frataxin inactivation, although leading to strong cardiac dysfunctions, did not reduce the adult longevity either (Fig. S8B). Overall, these results suggest that sarcomeric disorganization was mainly due to defective sarcomeric assembly during the cardiomyocyte maturation process.

### Prevention of histological defects by paclitaxel and MB treatments

Next, we evaluated the efficiency of paclitaxel and MB to prevent these histological defects. Both MB treatment (30 µM) and paclitaxel treatment (10 µM) restored the striated pattern of the actin network (Figs 3–5). Myosin striations and Sls sarcomeric pattern were also strongly improved, although some diffuse MHC-GFP and Sls-GFP staining were still detectable following paclitaxel treatment (Figs 3, 4). Thus, the effects of these two compounds on cardiomyocyte structural defects were correlated with their respective efficacy on functional cardiac parameters, as observed in Fig. 2. Then, we analyzed their effects on the MT network. As expected, paclitaxel treatment slightly increased the density of MTs in control *HandGS*/*+* flies. In frataxin-deficient *UAS-fhIR*/*+; Hand-GS*/*+*flies, the MT network was restored by paclitaxel treatment. Interestingly, MB treatment also led to a MT network similar to control hearts. Thus, paclitaxel and MB are both able to prevent structural and functional defects of frataxin-deficient cardiomyocytes.

## DISCUSSION

In this study, we used a cardiac model of FA in *Drosophila* to screen a medium-sized chemical library. To our knowledge, this is the first pharmacological screening of this extent performed *in vivo* on an animal model of cardiac disease and at the adult stage. Our study shows the feasibility of such a strategy, which can be carried out in a relatively short time (it took us around 12 months to achieve the primary screen) and at relatively low cost.

The Prestwick Chemical Library that we used here is composed of 1280 small molecules, all approved by the US Food and Drug Administration (FDA), European Medicines Agency (EMEA) or other agencies, and selected for their high chemical and pharmacological diversity, as well as for their known bioavailability and safety in humans. The choice of this type of chemical library was justified by the fact that the identification of protective compounds can lead to clinical applications rapidly by drug repositioning, which is particularly relevant in the case of rare diseases.

The compound with the strongest effects on cardiac dilatation was paclitaxel, an MT-stabilizing drug. Owing to its potent anti-mitotic properties, paclitaxel has been extensively used for the treatment of cancers, in particular ovarian, breast and lung cancers (Jordan and Wilson, 2004). It has also been evaluated on cellular and animal models in other pathological contexts, such as myocardial infarction and neurodegenerative diseases (Baas and Ahmad, 2013; Rodríguez-Sinovas et al., 2015; Xiao et al., 2011). We do not consider this drug as a candidate for therapeutic use in FA due to its toxicity, leading to a narrow therapeutic window. However, the identification of its protective effect in this screen is highly informative on the potent physiopathological mechanisms involved in FA cardiomyopathy, as discussed below. Apart from paclitaxel, ten compounds significantly reduced cardiac dilatation in our screen. Three of them have described effects on the cardiovascular system: sulmazole, a cardiotonic agent increasing cAMP (Endoh et al., 1985; Parsons et al., 1988); alfusozin, an α-adrenergic blocker, originally developed as an antihypertensive agent but now used as a treatment for benign prostatic hyperplasia (Roehrborn and Rosen, 2008); and bisoprolol fumarate, a β-blocker used in the treatment of hypertension and chronic heart failure. Indeed, bisoprolol is already recommended for slowing or preventing the deterioration of LV contraction in FA patients with reduced ejection fraction (Corben et al., 2014). Fluvoxamine, a selective serotonin reuptake inhibitor used as an antidepressant, was also selected in the screen. This compound is particularly interesting since it attenuated myocardial hypertrophy and the impaired LV FS induced by transverse aortic constriction in mice (Tagashira et al., 2010). Moreover, fluvoxamine improved mitochondrial Ca^2+^ influx and ATP production in neonatal rat hypertrophic cardiomyocytes. The proposed mechanism for this cardioprotective effect was through σ1-receptor stimulation (Tagashira et al., 2014). A cellular FA model based on frataxin silencing in human neuroblastoma cells showed that frataxin deficiency affected mitochondrial Ca^2+^ uptake capacity and reduced ATP production (Bolinches-Amoros et al., 2014). Thus, an attractive hypothesis would be that the protective effect of fluvoxamine in our cardiac model relies on its capacity to improve these mitochondrial dysfunctions. However, since there is no σ1-receptor described in *Drosophila*, whether fluvoxamine could have such effects on mitochondrial function in *Drosophila* should be further evaluated to validate this hypothesis.

Two other compounds have anti-inflammatory properties: flumethasone pivalate is a topical corticosteroid ester used in dermatology. Sulfasalazine is an anti-inflammatory drug used noticeably against inflammatory bowel diseases. Its metabolic breakdown product, 5-aminosalicylic acid (5-ASA), is a PPAR-γ agonist (Rousseaux et al., 2005). Actually, another PPAR-γ agonist, azeaolyl PAF, has the ability to increase the expression of frataxin in human neuroblastoma cells, and in primary fibroblasts from FA patients and from healthy controls (Marmolino et al., 2009). Therefore, an effect on frataxin expression level could be a potent mechanism for the protective effect of sulfasalazine detected here. The other compounds do not have documented properties or mechanisms of action with evident links to cardiac physiology or to known consequences of frataxin depletion. Two of them have significant toxic effects in humans and are therefore of little therapeutic interest: phenylpropanolamine, a synthetic sympathomimetic amine used noticeably as a weight loss agent, has been associated with hemorrhagic strokes and cerebral vasculitis, as well as infarctions (Yen and Ewald, 2012); and ethambutol, an antibacterial compound used in the treatment of tuberculosis, induces optic neuropathy in about 2% of treated patients (Sadun and Wang, 2008) and leads to mitochondrial toxicity, namely mitochondrial coupling defect and increased fragmentation of the mitochondrial network (Guillet et al., 2010). The two last compounds are mefloquine hydrochloride, an antimalarial agent, and ethotoin, a hydantoin derivative with anticonvulsant properties, used in the treatment of epilepsy. Therefore, among these eleven compounds, sulfasalazine and fluvoxamine appear to us to be the more relevant for future investigations. It should be noted, however, that their effects are relatively small compared to those of paclitaxel and MB, and thus should be confirmed on other models of the disease prior to extensive additional studies.

Oxidative stress has been proposed to play a central role in FA disease. However, the Prestwick Chemical Library contains several other molecules with antioxidant property considered as potent antioxidant medications for FA (pioglitazone, N-acetylcystein and seleginine), but none of them were selected in the screen. We have also previously shown that catalase overexpression or treatments with idebenone, a synthetic analog of coenzyme Q10 acting as a free-radical scavenger, or with the synthetic superoxide dismutase and catalase mimetic EUK8, failed to prevent cardiac dilatation or defective systolic function in fhIR flies (Tricoire et al., 2014). Similarly, MnTBAP, a MnSOD mimetic, had no beneficial effect on cardiomyopathy in a mouse model of FA (Seznec et al., 2005). Altogether, this suggests that, at least in flies and mice, oxidative stress is not a major contributor to the heart phenotypes induced by frataxin deficiency. Accordingly, clinical trials have not yet been able to show a clear effect of antioxidant compounds on the progression of the disease (Kearney et al., 2016).

We also identified six drugs that worsened the heart phenotype. These drugs did not induce heart dilatation in wild-type flies, showing that they were cardiotoxic specifically in a context of frataxin deficiency. They are used for various clinical applications: latanoprost is a prostaglandin analog, used in ophthalmic solutions to treat glaucoma. Zaleplon is a benzodiazepine receptor agonist and hypnotic used for the management of insomnia. Alosetron is a potent and selective 5-HT3 receptor antagonist that decreases gastrointestinal contraction and motility and gastrointestinal secretions. Ipriflavone is a synthetic isoflavone derivative used to treat osteoporosis. Finally, benfotiamine is a derivative of thiamine (vitamin B1), preventing advanced glycation end-product formation and is used in cases of diabetic neuropathy (Hosseini and Abdollahi, 2013). Noticeably, increased prevalence of osteoporosis and diabetes have been reported in FA patients (Cnop et al., 2013; Eigentler et al., 2014). Therefore, the existence of FA-specific potential cardiac adverse effects could be informative for clinicians, since these drugs might be prescribed to FA patients for therapeutic indications that are related or unrelated to the FA disease.

In the course of this study, we have also characterized the effect of frataxin deficiency on the sarcomeric organization of cardiomyocytes. We observed actin filaments with a lack of striated organization, but which were associated with a periodic pattern of the Z-disc Sls protein and partial myosin striation. This is reminiscent of nascent myofibrils proposed by Sanger et al. to be the step between premyofibrils and mature myofibrils during the myofibrillogenesis process in vertebrate striated muscles (Sanger et al., 2005, 2010). Therefore, our results suggest that frataxin deficiency leads to improper myofibril maturation, with defective sarcomere assembly in cardiomyocytes. In agreement with this hypothesis, frataxin inactivation at the adult stage in mature cardiomyocytes did not impair sarcomeric organization nor affect cardiac function. Thus, our results provide a new potent mechanism involved in the physiopathology of FA cardiomyopathy. In humans, cardiomyocyte proliferation occurs in children and adolescents up to the age of 20 years, and contributes to heart growth. After this age, cardiomyocyte proliferation seems either to stop or to gradually decrease, with estimations of renewal rates varying in different studies (Bergmann et al., 2009; Kajstura et al., 2010; Mollova et al., 2013). Interestingly, a study performed on 205 FA patients showed that the severity of the cardiomyopathy was not correlated to the GAA expansion length in these patients, but instead to the age at onset of the disease: patients with an early disease onset (lower than 14 years) had a more severe cardiac involvement than patients diagnosed later (Weidemann et al., 2012). In light of our results, this might be due to the specific requirement of frataxin in young humans for proper assembly of sarcomeres in maturing cardiomyocytes.

We also observed a full disruption of the MT network in frataxin-deficient *Drosophila* hearts. To our knowledge, the state of the MT network has not yet been studied in hearts of FA patients or in other cardiac models of the disease. However, a recent study showed that frataxin silencing alters MT stability in a motoneuronal cell line. In this study, frataxin deficiency also led to oxidative stress, to an increased pool of the GSSG/GSH (oxidized/reduced glutathione) ratio and to an increase of glutathionylated α-tubulin that were thought to be responsible for MT destabilization (Piermarini et al., 2016). Therefore, it appears that frataxin deficiency impacts the MT network in different cell types affected in the disease, although the mechanisms involved here in cardiomyocytes remain to be investigated.

The protective effect of paclitaxel shows that MT disruption is one of the main causes of cardiomyocyte dysfunction, at least in our *Drosophila* model. Indeed, the MT network has been proposed to be required for the positioning of myosin filaments during sarcomere formation in cultured skeletal myoblasts (Pizon et al., 2005). Consequently, the apparent improper myofibril maturation observed here could be a direct consequence of MT disruption. MTs are also required in beating cardiomyocytes to maintain their shape and organization, to resist compression and to transmit cellular signals by mechanotransduction (Robison and Prosser, 2017). Therefore, MT destabilization by frataxin deficiency could also affect various mechanical properties of matured cardiomyocytes. However, since treatment at 10 µM only allowed a partial rescue and we could not test this compound at higher doses because of its toxicity, it is very likely that other mechanisms besides MT network disorganization also contribute to heart dysfunction. Indeed, in various cellular and animal models, frataxin deficiency induces many cellular events, among which are mitochondrial dysfunction, perturbations of iron, lipid and calcium homeostasis, and oxidative and endoplasmic reticulum stress. Understanding the causal relationships between all these cellular events (including MT destabilization) and their respective involvement in functional defects remains a major challenge in the FA field.

Finally, our study confirmed the cardioprotective role of MB in frataxin-deficient hearts. We had already shown its efficacy on cardiac functional parameters (Tricoire et al., 2014); here, we show that it is also able to fully prevent sarcomeric disorganization in cardiomyocytes. Interestingly, the MT network was also rescued by MB treatment. Since this compound can function as an alternative electron carrier in mitochondria, we previously proposed that MB is protective through enhanced activity of the mitochondrial respiratory chain, which is known to be affected by frataxin deficiency (Bradley et al., 2000; Puccio et al., 2001; Rötig et al., 1997). It would be interesting to investigate whether the preserved actin and MT networks in MB-treated flies were also due to this property.

In conclusion, this pharmacological screen led to the identification of 11 drugs that significantly reduced heart dilatation of frataxin-depleted hearts. This study may, in the future, lead to therapeutic applications and improves our knowledge of the mechanisms involved in cardiac dysfunction associated with FA disease. In particular, it suggests that decreased contractility and dilatation of frataxin-depleted hearts are, at least in part, a consequence of defective sarcomeric assembly due to MT destabilization. More generally, our data highlight the power of *Drosophila* models of cardiac diseases for pharmacological approaches. We show here that it is feasible to perform pharmacological screens *in vivo* on a relatively large scale, under physiological conditions and using relevant functional parameters as readouts. This type of approach could therefore be extended in the future to a wide panel of cardiac diseases.

## MATERIALS AND METHODS

### *Drosophila* stocks, culture methods and treatment with compounds

UAS-fhIR (*w[1]; Pw[+mC]=UAS-fh.IR2)*, UAS-mitoGFP (*w[1118]; Pw[+mC]=UAS-mitoGFP.AP2/CyO*) and MHC:GFP (*y1 w; PBac{HpaI-GFP.A}MhcYD0783*) *Drosophila melanogaster* lines were obtained from the Bloomington Stock Center. The Sls-GFP line was kindly provided by John Sparrow (University of York, UK). The Hand-GS and daGS GeneSwitch lines were described in Monnier et al. (2012) and Tricoire et al. (2009), respectively. The fly food medium contained 60 g/l yeast extract, 34 g/l cornmeal, 50 g/l sucrose, 14 g/l agarose low gelling temperature (Sigma) and 25 ml/l methyl 4-hydroxybenzoate (200 g/l in ethanol). Drugs, provided by the Prestwick Company (Illkirch, France), were stored at −80°C in DMSO at a concentration of 10 mM and incorporated in the food medium at 37°C, at a final concentration of 10 µM. RU486 (Beta Pharma, Shanghai, China) was incorporated in the medium from a 20 mg/ml stock solution in ethanol at a final concentration of 40 ng/ml during development and 100 µg/ml during adulthood. For the primary screen, F0 flies were allowed to lay eggs in tubes containing the drugs and RU486 at 26°C. All untreated controls received an equivalent amount of DMSO (Sigma). After 10 days of development, adult F1 flies were collected within 24 h of eclosion under brief CO_2_ anesthesia, housed in groups of 20 under a 12 h-12 h light-dark cycle and transferred every 2 days onto fresh food medium containing RU486 (but not the drugs) prior to cardiac imaging, which was performed on 4- to 6-day-old males. Similar treatments and culture methods were used for validation experiments. Paclitaxel (semi-synthetic, Sigma) and MB (Sigma) were used for dose-response assays and histological studies.

### *In vivo* imaging of fly hearts and movie analysis

Flies were anesthetized with FlyNAP (Carolina Biological Supply Company). The anterior parts of heart (abdominal segments A1/A2) were observed with a Zeiss SteREO Lumar.V12 stereomicroscope, with a NeoLumar S 1.5× objective. Video movies were acquired with a Hamamastu Orca Flash 4.0 LT camera (50 frames per second, 501 frames per movie). For the primary screen, each video was analyzed as described in Seguin et al. (2015) using ImageJ to estimate the diastolic diameter (DD), from a picture generated by flattening all frames into one (code available at https://github.com/MichaelRera/autoMmodeGen). This method was used at this step because it was appropriate and fast enough to analyze several thousand movies, as required during the primary screen. For each compound (Cn), the ICD (index of cardiac dilatation) was then calculated as follows:
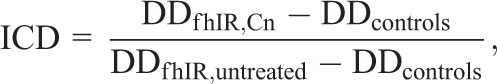
where DD is the median values obtained for all flies of the same genotype and the same treatment. For validation experiments, we used the analysis method described in Monnier et al. (2012) that allowed to extract more cardiac functional parameters, noticeably EDD, ESD and FS. FS was calculated as described in Fink et al. (2009). On the same principle as ICD, we calculated indexes of diastolic and systolic dilatation (IDD and ISD, respectively) as follows:
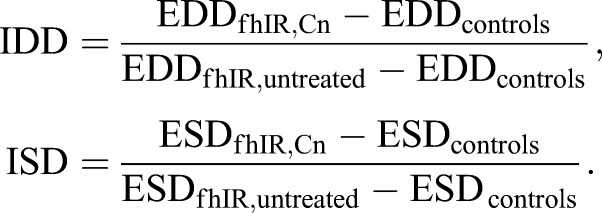


### Statistical analysis

For selection of drugs in the primary screen, comparison of DDs of treated and untreated fhIR flies were carried out independently for each drug subset to ensure that control flies experienced the same conditions as drug-treated flies. Analysis was performed with R (https://r-project.org) under RStudio environment (https://rstudio.com) and the statistical significance assessed by non-parametric Wilcoxon analysis. Additionally, a global analysis was performed at the end of the whole screen on normalized data, and compounds corresponding to a *Z*-score >2 were added to the initial selection for further validation.

For the validation step, since several experiments were performed on each selected compound, we included all the experimental values in an Access database to facilitate subsequent analysis. To take into account small variations occurring between independent experiments, we first normalized diastolic and systolic diameters in each experiment (*i*) with a coefficient *N*_i_ calculated as:
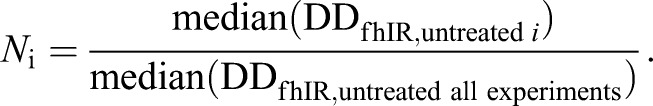
This procedure allowed us to perform a statistical analysis of all these normalized values with the GraphPad Prism 6 software. Statistical significance of each selected compound was assessed with non-parametric ANOVA (Kruskal–Wallis test with Dunn's *post hoc* test) and compounds with *P-*values <5×10^−2^ were retained.

### Immunostaining of adult *Drosophila* hearts

Dissection and immunostaining were performed as described in Monier et al. (2005), except for MT staining, for which heart dissections and fixations were performed in BRB80 (80 mM PIPES, pH 6.8, 1 mM MgCl_2_, 1 mM EGTA) as described in Legent et al. (2015). Seven to twelve hearts were dissected for each independent condition. The following primary antibodies were used: rabbit anti-GFP (TP401, Torrey Pines), used at 1/500, and mouse anti α-tubulin (clone B512, Sigma), used at 1/750. The secondary antibodies donkey anti-mouse and goat anti-rabbit conjugated with Alexa Fluor 488 dye (ThermoFisher Scientific) were used at 1/500. F-actin was stained with phalloidin–Atto 647N (Sigma). Samples were mounted onto slides in ProlongGold ProLong™ Gold Antifade Mountant (ThermoFisher Scientific). Images were acquired with a Zeiss LSM-710 microscope. Images were processed using Fiji.

### qPCR

To measure *fh* mRNA levels in whole larvae, five samples of ten wandering third-instar larvae were collected. Total RNA was extracted as described in Reinhardt et al. (2012). First-strand cDNA was synthesized from 3 µg total RNA with Superscript III (Invitrogen). Quantitative real-time PCR (qPCR) analysis was performed on a LightCycler 480 with SYBR Green labeling. The *RpL32* gene was used as a reference. The primers used for *fh* amplification were DFH51: 5′-ACACCCTGGACGCACTGT-3′ and DFH31: 5′-CCAGGTTCACGGTTAGCAC-3′.

### Lifespan analysis

Flies were collected within 24 h of eclosion under brief CO_2_ anesthesia, housed in groups of 30, and raised at 26°C under a 12 h-12 h light-dark cycle. RU486 treatment when applied was 1 µg/ml during development and 100 µg/ml during adulthood. Flies were transferred every 2 days onto fresh food, and dead flies were counted.

## Supplementary Material

Supplementary information

## References

[DMM033811C1] Anderson P. R., Kirby K., Hilliker A. J. and Phillips J. P. (2005). RNAi-mediated suppression of the mitochondrial iron chaperone, frataxin, in Drosophila. *Hum. Mol. Genet.* 14, 3397-3405. 10.1093/hmg/ddi367 16203742

[DMM033811C2] Anderson P. R., Kirby K., Orr W. C., Hilliker A. J. and Phillips J. P. (2008). Hydrogen peroxide scavenging rescues frataxin deficiency in a Drosophila model of Friedreich's ataxia. *Proc. Natl. Acad. Sci. USA* 105, 611-616. 10.1073/pnas.0709691105 18184803 PMC2206584

[DMM033811C3] Aranca T. V., Jones T. M., Shaw J. D., Staffetti J. S., Ashizawa T., Kuo S.-H., Fogel B. L., Wilmot G. R., Perlman S. L., Onyike C. U. et al. (2016). Emerging therapies in Friedreich's ataxia. *Neurodegener. Dis. Manag.* 6, 49-65. 10.2217/nmt.15.73 26782317 PMC4768799

[DMM033811C4] Baas P. W. and Ahmad F. J. (2013). Beyond taxol: microtubule-based treatment of disease and injury of the nervous system. *Brain* 136, 2937-2951. 10.1093/brain/awt153 23811322 PMC3784279

[DMM033811C5] Babcock M., de Silva D., Oaks R., Davis-Kaplan S., Jiralerspong S., Montermini L., Pandolfo M. and Kaplan J. (1997). Regulation of mitochondrial iron accumulation by Yfh1p, a putative homolog of frataxin. *Science* 276, 1709-1712. 10.1126/science.276.5319.1709 9180083

[DMM033811C6] Bergmann O., Bhardwaj R. D., Bernard S., Zdunek S., Barnabé-Heider F., Walsh S., Zupicich J., Alkass K., Buchholz B. A., Druid H. et al. (2009). Evidence for cardiomyocyte renewal in humans. *Science* 324, 98-102. 10.1126/science.1164680 19342590 PMC2991140

[DMM033811C7] Bolinches-Amoros A., Molla B., Pla-Martin D., Palau F. and Gonzalez-Cabo P. (2014). Mitochondrial dysfunction induced by frataxin deficiency is associated with cellular senescence and abnormal calcium metabolism. *Front. Cell Neurosci.* 8, 124. 24860428 10.3389/fncel.2014.00124 PMC4026758

[DMM033811C8] Bradley J. L., Blake J. C., Chamberlain S., Thomas P. K., Cooper J. M. and Schapira A. H. (2000). Clinical, biochemical and molecular genetic correlations in Friedreich's ataxia. *Hum. Mol. Genet.* 9, 275-282. 10.1093/hmg/9.2.275 10607838

[DMM033811C9] Braymer J. J. and Lill R. (2017). Iron-sulfur cluster biogenesis and trafficking in mitochondria. *J. Biol. Chem.* 292, 12754-12763. 10.1074/jbc.R117.787101 28615445 PMC5546016

[DMM033811C10] Burkart C., Qiu F., Brendel S., Benes V., Hååg P., Labeit S., Leonard K. and Bullard B. (2007). Modular proteins from the Drosophila sallimus (sls) gene and their expression in muscles with different extensibility. *J. Mol. Biol.* 367, 953-969. 10.1016/j.jmb.2007.01.059 17316686

[DMM033811C11] Calmels N., Seznec H., Villa P., Reutenauer L., Hibert M., Haiech J., Rustin P., Koenig M. and Puccio H. (2009). Limitations in a frataxin knockdown cell model for Friedreich ataxia in a high-throughput drug screen. *BMC Neurol.* 9, 46 10.1186/1471-2377-9-46 19703283 PMC2744904

[DMM033811C12] Campuzano V., Montermini L., Molto M. D., Pianese L., Cossee M., Cavalcanti F., Monros E., Rodius F., Duclos F., Monticelli A. et al. (1996). Friedreich's ataxia: autosomal recessive disease caused by an intronic GAA triplet repeat expansion. *Science* 271, 1423-1427. 10.1126/science.271.5254.1423 8596916

[DMM033811C13] Casazza F. and Morpurgo M. (1996). The varying evolution of Friedreich's ataxia cardiomyopathy. *Am. J. Cardiol.* 77, 895-898. 10.1016/S0002-9149(97)89194-1 8623752

[DMM033811C14] Child J. S., Perloff J. K., Bach P. M., Wolfe A. D., Perlman S. and Pieter Kark R. A. (1986). Cardiac involvement in Friedreich's ataxia: a clinical study of 75 patients. *J. Am. Coll. Cardiol.* 7, 1370-1378. 10.1016/S0735-1097(86)80159-0 2940284

[DMM033811C15] Cnop M., Mulder H. and Igoillo-Esteve M. (2013). Diabetes in Friedreich ataxia. *J. Neurochem.* 126 Suppl. 1, 94-102. 10.1111/jnc.12216 23859345

[DMM033811C16] Corben L. A., Lynch D., Pandolfo M., Schulz J. B. and Delatycki M. B. (2014). Consensus clinical management guidelines for Friedreich ataxia. *Orphanet J. Rare Dis.* 9, 184 10.1186/s13023-014-0184-7 25928624 PMC4280001

[DMM033811C17] Cotticelli M. G., Rasmussen L., Kushner N. L., McKellip S., Sosa M. I., Manouvakhova A., Feng S., White E. L., Maddry J. A., Heemskerk J. et al. (2012). Primary and secondary drug screening assays for Friedreich ataxia. *J. Biomol. Screen.* 17, 303-313. 10.1177/1087057111427949 22086726

[DMM033811C18] Delatycki M. B., Williamson R. and Forrest S. M. (2000). Friedreich ataxia: an overview. *J. Med. Genet.* 37, 1-8. 10.1136/jmg.37.1.1 10633128 PMC1734457

[DMM033811C19] Dürr A., Cossee M., Agid Y., Campuzano V., Mignard C., Penet C., Mandel J.-L., Brice A. and Koenig M. (1996). Clinical and genetic abnormalities in patients with Friedreich's ataxia. *N. Engl. J. Med.* 335, 1169-1175. 10.1056/NEJM199610173351601 8815938

[DMM033811C20] Dutka D. P., Donnelly J. E., Palka P., Lange A., Nunez D. J. R. and Nihoyannopoulos P. (2000). Echocardiographic characterization of cardiomyopathy in Friedreich's ataxia with tissue Doppler echocardiographically derived myocardial velocity gradients. *Circulation* 102, 1276-1282. 10.1161/01.CIR.102.11.1276 10982543

[DMM033811C21] Eigentler A., Nachbauer W., Donnemiller E., Poewe W., Gasser R. W. and Boesch S. (2014). Low bone mineral density in Friedreich ataxia. *Cerebellum* 13, 549-557. 10.1007/s12311-014-0568-1 24858524

[DMM033811C22] Endoh M., Yanagisawa T., Morita T. and Taira N. (1985). Differential effects of sulmazole (AR-L 115 BS) on contractile force and cyclic AMP levels in canine ventricular muscle: comparison with MDL 17,043. *J. Pharmacol. Exp. Ther.* 234, 267-273. 2989507

[DMM033811C23] Fink M., Callol-Massot C., Chu A., Ruiz-Lozano P., Izpisua Belmonte J. C. I., Giles W., Bodmer R. and Ocorr K. (2009). A new method for detection and quantification of heartbeat parameters in Drosophila, zebrafish, and embryonic mouse hearts. *BioTechniques* 46, 101-113. 10.2144/000113078 19317655 PMC2855226

[DMM033811C24] Giunta A., Maione S., Biagini R., Filla A., De Michele G. and Campanella G. (1988). Noninvasive assessment of systolic and diastolic function in 50 patients with Friedreich's ataxia. *Cardiology* 75, 321-327. 10.1159/000174394 3233613

[DMM033811C25] Guillet V., Chevrollier A., Cassereau J., Letournel F., Gueguen N., Richard L., Desquiret V., Verny C., Procaccio V., Amati-Bonneau P. et al. (2010). Ethambutol-induced optic neuropathy linked to OPA1 mutation and mitochondrial toxicity. *Mitochondrion* 10, 115-124. 10.1016/j.mito.2009.11.004 19900585

[DMM033811C26] Harding A. E. (1981). Friedreich's ataxia: a clinical and genetic study of 90 families with an analysis of early diagnostic criteria and intrafamilial clustering of clinical features. *Brain* 104, 589-620. 10.1093/brain/104.3.589 7272714

[DMM033811C27] Hawley R. J. and Gottdiener J. S. (1986). Five-year follow-up of Friedreich's ataxia cardiomyopathy. *Arch. Intern. Med.* 146, 483-488. 10.1001/archinte.1986.00360150081010 3954519

[DMM033811C28] Hosseini A. and Abdollahi M. (2013). Diabetic neuropathy and oxidative stress: therapeutic perspectives. *Oxid. Med. Cell Longev.* 2013, 168039 10.1155/2013/168039 23738033 PMC3655656

[DMM033811C29] Huang M. L.-H., Becker E. M., Whitnall M., Suryo Rahmanto Y., Ponka P. and Richardson D. R. (2009). Elucidation of the mechanism of mitochondrial iron loading in Friedreich's ataxia by analysis of a mouse mutant. *Proc. Natl. Acad. Sci. USA* 106, 16381-16386. 10.1073/pnas.0906784106 19805308 PMC2752539

[DMM033811C30] Jordan M. A. and Wilson L. (2004). Microtubules as a target for anticancer drugs. *Nat. Rev. Cancer* 4, 253-265. 10.1038/nrc1317 15057285

[DMM033811C31] Kajstura J., Urbanek K., Perl S., Hosoda T., Zheng H., Ogorek B., Ferreira-Martins J., Goichberg P., Rondon-Clavo C., Sanada F. et al. (2010). Cardiomyogenesis in the adult human heart. *Circ. Res.* 107, 305-315. 10.1161/CIRCRESAHA.110.223024 20522802 PMC2987602

[DMM033811C32] Kearney M., Orrell R. W., Fahey M., Brassington R. and Pandolfo M. (2016). Pharmacological treatments for Friedreich ataxia. *Cochrane Database Syst. Rev.* 8, CD007791 10.1002/14651858.CD007791.pub4 PMC6457808 27572719

[DMM033811C33] Kipps A., Alexander M., Colan S. D., Gauvreau K., Smoot L., Crawford L., Darras B. T. and Blume E. D. (2009). The longitudinal course of cardiomyopathy in Friedreich's ataxia during childhood. *Pediatr. Cardiol.* 30, 306-310. 10.1007/s00246-008-9305-1 18716706

[DMM033811C34] Koeppen A. H. (2011). Friedreich's ataxia: pathology, pathogenesis, and molecular genetics. *J. Neurol. Sci.* 303, 1-12. 10.1016/j.jns.2011.01.010 21315377 PMC3062632

[DMM033811C35] Legent K., Tissot N. and Guichet A. (2015). Visualizing microtubule networks during Drosophila oogenesis using fixed and live imaging. *Methods Mol. Biol.* 1328, 99-112. 10.1007/978-1-4939-2851-4_7 26324432

[DMM033811C36] Llorens J. V., Navarro J. A., Martínez-Sebastián M. J., Baylies M. K., Schneuwly S., Botella J. A. and Moltó M. D. (2007). Causative role of oxidative stress in a Drosophila model of Friedreich ataxia. *FASEB J.* 21, 333-344. 10.1096/fj.05-5709com 17167074

[DMM033811C37] Marmolino D., Acquaviva F., Pinelli M., Monticelli A., Castaldo I., Filla A. and Cocozza S. (2009). PPAR-gamma agonist Azelaoyl PAF increases frataxin protein and mRNA expression: new implications for the Friedreich's ataxia therapy. *Cerebellum* 8, 98-103. 10.1007/s12311-008-0087-z 19104905

[DMM033811C38] Mollova M., Bersell K., Walsh S., Savla J., Das L. T., Park S.-Y., Silberstein L. E., dos Remedios C. G., Graham D., Colan S. et al. (2013). Cardiomyocyte proliferation contributes to heart growth in young humans. *Proc. Natl. Acad. Sci. USA* 110, 1446-1451. 10.1073/pnas.1214608110 23302686 PMC3557060

[DMM033811C39] Monier B., Astier M., Semeriva M. and Perrin L. (2005). Steroid-dependent modification of Hox function drives myocyte reprogramming in the Drosophila heart. *Development* 132, 5283-5293. 10.1242/dev.02091 16284119

[DMM033811C40] Monnier V., Iché-Torres M., Rera M., Contremoulins V., Guichard C., Lalevée N., Tricoire H. and Perrin L. (2012). dJun and Vri/dNFIL3 are major regulators of cardiac aging in Drosophila. *PLoS Genet.* 8, e1003081 10.1371/journal.pgen.1003081 23209438 PMC3510041

[DMM033811C41] Morin X., Daneman R., Zavortink M. and Chia W. (2001). A protein trap strategy to detect GFP-tagged proteins expressed from their endogenous loci in Drosophila. *Proc. Natl. Acad. Sci. USA* 98, 15050-15055. 10.1073/pnas.261408198 11742088 PMC64981

[DMM033811C42] Morvan D., Komajda M., Doan L. D., Brice A., Isnard R., Seck R., Lechat P., Agid Y. and Grosgogeat Y. (1992). Cardiomyopathy in Friedreich's ataxia: a Doppler-echocardiographic study. *Eur. Heart J.* 13, 1393-1398. 10.1093/oxfordjournals.eurheartj.a060072 1396814

[DMM033811C43] Navarro J. A., Ohmann E., Sanchez D., Botella J. A., Liebisch G., Molto M. D., Ganfornina M. D., Schmitz G. and Schneuwly S. (2010). Altered lipid metabolism in a Drosophila model of Friedreich's ataxia. *Hum. Mol. Genet.* 19, 2828-2840. 10.1093/hmg/ddq183 20460268 PMC7108586

[DMM033811C44] Orfanos Z., Leonard K., Elliott C., Katzemich A., Bullard B. and Sparrow J. (2015). Sallimus and the dynamics of sarcomere assembly in Drosophila flight muscles. *J. Mol. Biol.* 427, 2151-2158. 10.1016/j.jmb.2015.04.003 25868382

[DMM033811C45] Pandolfo M. (2009). Friedreich ataxia: the clinical picture. *J. Neurol.* 256 Suppl. 1, 3-8. 10.1007/s00415-009-1002-3 19283344

[DMM033811C46] Parsons W. J., Ramkumar V. and Stiles G. L. (1988). The new cardiotonic agent sulmazole is an A1 adenosine receptor antagonist and functionally blocks the inhibitory regulator, Gi. *Mol. Pharmacol.* 33, 441-448. 3128727

[DMM033811C47] Piermarini E., Cartelli D., Pastore A., Tozzi G., Compagnucci C., Giorda E., D'Amico J., Petrini S., Bertini E., Cappelletti G. et al. (2016). Frataxin silencing alters microtubule stability in motor neurons: implications for Friedreich's ataxia. *Hum. Mol. Genet.* 25, 4288-4301. 10.1093/hmg/ddw260 27516386

[DMM033811C48] Pizon V., Gerbal F., Diaz C. C. and Karsenti E. (2005). Microtubule-dependent transport and organization of sarcomeric myosin during skeletal muscle differentiation. *EMBO J.* 24, 3781-3792. 10.1038/sj.emboj.7600842 16237460 PMC1276724

[DMM033811C49] Puccio H., Simon D., Cossée M., Criqui-Filipe P., Tiziano F., Melki J., Hindelang C., Matyas R., Rustin P. and Koenig M. (2001). Mouse models for Friedreich ataxia exhibit cardiomyopathy, sensory nerve defect and Fe-S enzyme deficiency followed by intramitochondrial iron deposits. *Nat. Genet.* 27, 181-186. 10.1038/84818 11175786

[DMM033811C50] Raman S. V., Phatak K., Hoyle J. C., Pennell M. L., McCarthy B., Tran T., Prior T. W., Olesik J. W., Lutton A., Rankin C. et al. (2011). Impaired myocardial perfusion reserve and fibrosis in Friedreich ataxia: a mitochondrial cardiomyopathy with metabolic syndrome. *Eur. Heart J.* 32, 561-567. 10.1093/eurheartj/ehq443 21156720 PMC3106287

[DMM033811C51] Regner S. R., Lagedrost S. J., Plappert T., Paulsen E. K., Friedman L. S., Snyder M. L., Perlman S. L., Mathews K. D., Wilmot G. R., Schadt K. A. et al. (2012). Analysis of echocardiograms in a large heterogeneous cohort of patients with friedreich ataxia. *Am. J. Cardiol.* 109, 401-405. 10.1016/j.amjcard.2011.09.025 22078220

[DMM033811C52] Reinhardt A., Feuillette S., Cassar M., Callens C., Thomassin H., Birman S., Lecourtois M., Antoniewski C. and Tricoire H. (2012). Lack of miRNA misregulation at early pathological stages in Drosophila neurodegenerative disease models. *Front. Genet.* 3, 226 10.3389/fgene.2012.00226 23115562 PMC3483601

[DMM033811C53] Robison P. and Prosser B. L. (2017). Microtubule mechanics in the working myocyte. *J. Physiol.* 595, 3931-3937. 10.1113/JP273046 28116814 PMC5471505

[DMM033811C54] Rodríguez-Sinovas A., Abad E., Sánchez J. A., Fernández-Sanz C., Inserte J., Ruiz-Meana M., Alburquerque-Béjar J. J. and García-Dorado D. (2015). Microtubule stabilization with paclitaxel does not protect against infarction in isolated rat hearts. *Exp. Physiol.* 100, 23-34. 10.1113/expphysiol.2014.082925 25557728

[DMM033811C55] Roehrborn C. G. and Rosen R. C. (2008). Medical therapy options for aging men with benign prostatic hyperplasia: focus on alfuzosin 10 mg once daily. *Clin. Interv. Aging* 3, 511-524. 10.2147/CIA.S3635 18982921 PMC2682383

[DMM033811C56] Rötig A., de Lonlay P., Chretien D., Foury F., Koenig M., Sidi D., Munnich A. and Rustin P. (1997). Aconitase and mitochondrial iron-sulphur protein deficiency in Friedreich ataxia. *Nat. Genet.* 17, 215-217. 10.1038/ng1097-215 9326946

[DMM033811C57] Rousseaux C., Lefebvre B., Dubuquoy L., Lefebvre P., Romano O., Auwerx J., Metzger D., Wahli W., Desvergne B., Naccari G. C. et al. (2005). Intestinal antiinflammatory effect of 5-aminosalicylic acid is dependent on peroxisome proliferator-activated receptor-gamma. *J. Exp. Med.* 201, 1205-1215. 10.1084/jem.20041948 15824083 PMC2213148

[DMM033811C58] Sadun A. A. and Wang M. Y. (2008). Ethambutol optic neuropathy: how we can prevent 100,000 new cases of blindness each year. *J. Neuroophthalmol.* 28, 265-268. 10.1097/WNO.0b013e31818f138f 19145122

[DMM033811C59] Sanger J. W., Kang S., Siebrands C. C., Freeman N., Du A., Wang J., Stout A. L. and Sanger J. M. (2005). How to build a myofibril. *J. Muscle Res. Cell Motil.* 26, 343-354. 10.1007/s10974-005-9016-7 16465476

[DMM033811C60] Sanger J. W., Wang J., Fan Y., White J. and Sanger J. M. (2010). Assembly and dynamics of myofibrils. *J. Biomed. Biotechnol.* 2010, 858606 10.1155/2010/858606 20625425 PMC2896905

[DMM033811C61] Seguin A., Monnier V., Palandri A., Bihel F., Rera M., Schmitt M., Camadro J.-M., Tricoire H. and Lesuisse E. (2015). A yeast/Drosophila screen to identify new compounds overcoming frataxin deficiency. *Oxid. Med. Cell Longev.* 2015, 565140 10.1155/2015/565140 26523199 PMC4619980

[DMM033811C62] Seznec H., Simon D., Monassier L., Criqui-Filipe P., Gansmuller A., Rustin P., Koenig M. and Puccio H. (2004). Idebenone delays the onset of cardiac functional alteration without correction of Fe-S enzymes deficit in a mouse model for Friedreich ataxia. *Hum. Mol. Genet.* 13, 1017-1024. 10.1093/hmg/ddh114 15028670

[DMM033811C63] Seznec H., Simon D., Bouton C., Reutenauer L., Hertzog A., Golik P., Procaccio V., Patel M., Drapier J.-C., Koenig M. et al. (2005). Friedreich ataxia: the oxidative stress paradox. *Hum. Mol. Genet.* 14, 463-474. 10.1093/hmg/ddi042 15615771

[DMM033811C64] Tagashira H., Bhuiyan S., Shioda N., Hasegawa H., Kanai H. and Fukunaga K. (2010). Sigma1-receptor stimulation with fluvoxamine ameliorates transverse aortic constriction-induced myocardial hypertrophy and dysfunction in mice. *Am. J. Physiol. Heart Circ. Physiol.* 299, H1535-H1545. 10.1152/ajpheart.00198.2010 20802134

[DMM033811C65] Tagashira H., Bhuiyan M. S., Shioda N. and Fukunaga K. (2014). Fluvoxamine rescues mitochondrial Ca2+ transport and ATP production through sigma(1)-receptor in hypertrophic cardiomyocytes. *Life Sci.* 95, 89-100. 10.1016/j.lfs.2013.12.019 24373833

[DMM033811C66] Tricoire H., Battisti V., Trannoy S., Lasbleiz C., Pret A.-M. and Monnier V. (2009). The steroid hormone receptor EcR finely modulates Drosophila lifespan during adulthood in a sex-specific manner. *Mech. Ageing Dev.* 130, 547-552. 10.1016/j.mad.2009.05.004 19486910

[DMM033811C67] Tricoire H., Palandri A., Bourdais A., Camadro J.-M. and Monnier V. (2014). Methylene blue rescues heart defects in a Drosophila model of Friedreich's ataxia. *Hum. Mol. Genet.* 23, 968-979. 10.1093/hmg/ddt493 24105471

[DMM033811C68] Tsou A. Y., Paulsen E. K., Lagedrost S. J., Perlman S. L., Mathews K. D., Wilmot G. R., Ravina B., Koeppen A. H. and Lynch D. R. (2011). Mortality in Friedreich ataxia. *J. Neurol. Sci.* 307, 46-49. 10.1016/j.jns.2011.05.023 21652007

[DMM033811C69] Unverferth D. V., Schmidt W. R. II, Baker P. B. and Wooley C. F. (1987). Morphologic and functional characteristics of the heart in Friedreich's ataxia. *Am. J. Med.* 82, 5-10. 10.1016/0002-9343(87)90369-X 3799693

[DMM033811C70] Weidemann F., Rummey C., Bijnens B., Stork S., Jasaityte R., Dhooge J., Baltabaeva A., Sutherland G., Schulz J. B. and Meier T. (2012). The heart in Friedreich ataxia: definition of cardiomyopathy, disease severity, and correlation with neurological symptoms. *Circulation* 125, 1626-1634. 10.1161/CIRCULATIONAHA.111.059477 22379112

[DMM033811C71] Whitnall M., Suryo Rahmanto Y., Sutak R., Xu X., Becker E. M., Mikhael M. R., Ponka P. and Richardson D. R. (2008). The MCK mouse heart model of Friedreich's ataxia: alterations in iron-regulated proteins and cardiac hypertrophy are limited by iron chelation. *Proc. Natl. Acad. Sci. USA* 105, 9757-9762. 10.1073/pnas.0804261105 18621680 PMC2474513

[DMM033811C72] Wong A., Yang J., Cavadini P., Gellera C., Lonnerdal B., Taroni F. and Cortopassi G. (1999). The Friedreich's ataxia mutation confers cellular sensitivity to oxidant stress which is rescued by chelators of iron and calcium and inhibitors of apoptosis. *Hum. Mol. Genet.* 8, 425-430. 10.1093/hmg/8.3.425 9949201

[DMM033811C73] Xiao J., Cao H., Liang D., Liu Y., Zhang H., Zhao H., Li J., Yan B., Peng L., Zhou Z. et al. (2011). Taxol, a microtubule stabilizer, prevents ischemic ventricular arrhythmias in rats. *J. Cell. Mol. Med.* 15, 1166-1176. 10.1111/j.1582-4934.2010.01106.x 20561109 PMC3822629

[DMM033811C74] Yen M. and Ewald M. B. (2012). Toxicity of weight loss agents. *J. Med. Toxicol.* 8, 145-152. 10.1007/s13181-012-0213-7 22351299 PMC3550246

