# Supplementary material for: Identification of cardioprotective drugs by medium-scale in vivo pharmacological screening on a Drosophila cardiac model of Friedreich's ataxia
Source: Dis Model Mech. 2018 Jul 20;11(7):dmm033811. doi: 10.1242/dmm.033811 (PMC6078405; doi:10.1242/dmm.033811)
Supplement: Supplementary information [file dmm-11-033811-s1.pdf]

Drug		Genotype	n	Diastolic diameter			Systolic diameter		
Name	Conc.			EDD (µm)	IDD	Statistical analysis	ESD (µm)	ISD	Statistical analysis
Untreated		+	569	56,1	0,00	***	29,6	0,00	***
**Untreated**		**fhIR**	**1502**	**101,6**	**1,00**		**71,3**	**1,00**	
**Sulfasalazine**	1µM	**fhIR**	**43**	**99,8**	**0,96**	**ns**	**63,7**	**0,82**	**ns**
	10µM	**fhIR**	**146**	**92,5**	**0,80**	**	**63,5**	**0,81**	*
	30µM	**fhIR**	**40**	**99,3**	**0,95**	**ns**	**61,8**	**0,77**	**ns**
**Mefloquine hydrochloride**	1µM	**fhIR**	**40**	**94,3**	**0,84**	*	**63,1**	**0,80**	*
	10µM	**fhIR**	**166**	**92,9**	**0,81**	***	**62,0**	**0,78**	***
	30µM	**fhIR**	**55**	**97,6**	**0,91**	*	**60,7**	**0,75**	**
**Flumethasone pivalate**	1µM	**fhIR**	**50**	**100,1**	**0,97**	**ns**	**67,2**	**0,90**	**ns**
	10µM	**fhIR**	**115**	**93,6**	**0,82**	**	**63,1**	**0,80**	**
	30µM	**fhIR**	**53**	**99,8**	**0,96**	**ns**	**68,6**	**0,94**	**ns**
**Phenylpropanolamine hydrochloride**	1µM	**fhIR**	**32**	**101,3**	**0,99**	**ns**	**67,0**	**0,90**	**ns**
	10µM	**fhIR**	**143**	**94,0**	**0,83**	*	**63,9**	**0,82**	*
	30µM	**fhIR**	**43**	**98,3**	**0,93**	**ns**	**63,1**	**0,80**	*
**Fluvoxamine maleate**	1µM	**fhIR**	**44**	**94,4**	**0,84**	**ns**	**68,1**	**0,92**	**ns**
	10µM	**fhIR**	**149**	**94,7**	**0,85**	***	**63,9**	**0,82**	*
	30µM	**fhIR**	**49**	**97,9**	**0,92**	**ns**	**68,6**	**0,93**	**ns**
**Sulmazole**	1µM	**fhIR**	**48**	**72,8**	**0,37**	***	**49,9**	**0,49**	***
	10µM	**fhIR**	**141**	**95,1**	**0,86**	*	**65,7**	**0,87**	**ns**
	30µM	**fhIR**	**51**	**95,5**	**0,86**	**ns**	**61,2**	**0,76**	*
**Alfuzosin hydrochloride**	1µM	**fhIR**	**40**	**97,6**	**0,91**	**ns**	**63,4**	**0,81**	*
	10µM	**fhIR**	**162**	**96,1**	**0,88**	**	**69,2**	**0,95**	**ns**
	30µM	**fhIR**	**42**	**103,4**	**1,04**	**ns**	**64,8**	**0,85**	**ns**
**Ethambutol dihydrochloride**	1µM	**fhIR**	**39**	**81,0**	**0,55**	***	**51,8**	**0,53**	***
	10µM	**fhIR**	**165**	**95,8**	**0,87**	*	**65,0**	**0,85**	*
	30µM	**fhIR**	**57**	**93,3**	**0,82**	**	**57,5**	**0,67**	***
**Bisoprolol fumarate**	1µM	**fhIR**	**37**	**102,4**	**1,02**	**ns**	**68,2**	**0,93**	**ns**
	10µM	**fhIR**	**128**	**95,8**	**0,87**	*	**65,4**	**0,86**	*
	30µM	**fhIR**	**39**	**102,0**	**1,01**	**ns**	**64,5**	**0,84**	**ns**
**Ethotoin**	1µM	**fhIR**	**46**	**92,2**	**0,79**	***	**62,1**	**0,78**	*
	10µM	**fhIR**	**180**	**95,8**	**0,87**	*	**66,9**	**0,89**	**ns**
	30µM	**fhIR**	**47**	**104,5**	**1,06**	**ns**	**72,3**	**1,02**	**ns**

Table S1 : Cardiac parameters of flies treated with protective drugs at different concentrations.

The drugs validated for their protective effect at 10 µM were tested at two additional concentrations, 1 µM and 30 µM. The values of EDD and ESD correspond to the median values obtained on a global analysis including all independent experiments. IDD and ISD were calculated as indicated in the Methods section. n: number of flies. Statistical significance was assessed independently for changes in EDD or ESD of fhIR flies treated with drugs compared to control fhIR untreated flies, with non-parametric Anova (Kruskal-Wallis test with Dunn's post hoc test) * $p<5.10^{-2}$. ** $p<1.10^{-3}$. *** $p<1.10^{-4}$. ns : not specific.

Disease Models & Mechanisms • Supplementary information

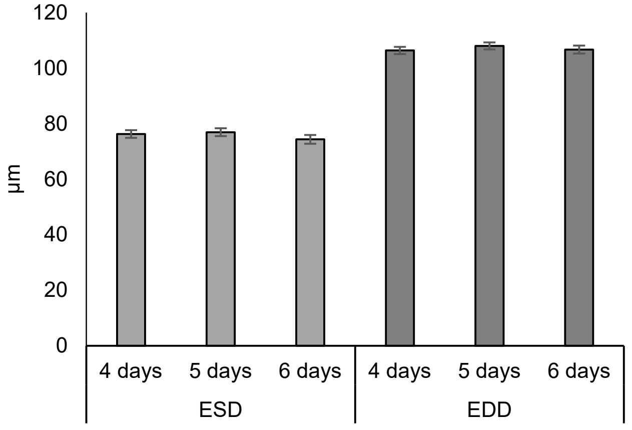

Figure S1 : Stability of the cardiac phenotype in 4 to 6 days old frataxin-deficient flies. End-Systolic Diameter (ESD, µm) and End-Diastolic Diameter (EDD, µm) of 4 days (n=132), 5 days (n=121) and 6 days old (n=118) *UAS-mitoGFP/UAS-fhIR; Hand-GS/+* (fhIR) male flies. All flies were fed with RU486 during both development (40 ng/ml of food) and adulthood (100 µg/ml). All values are means (±SEM). Statistical significance was assessed by non-parametric Wilcoxon analysis. No significant differences between ESD or EDD of flies of different ages could be detected.

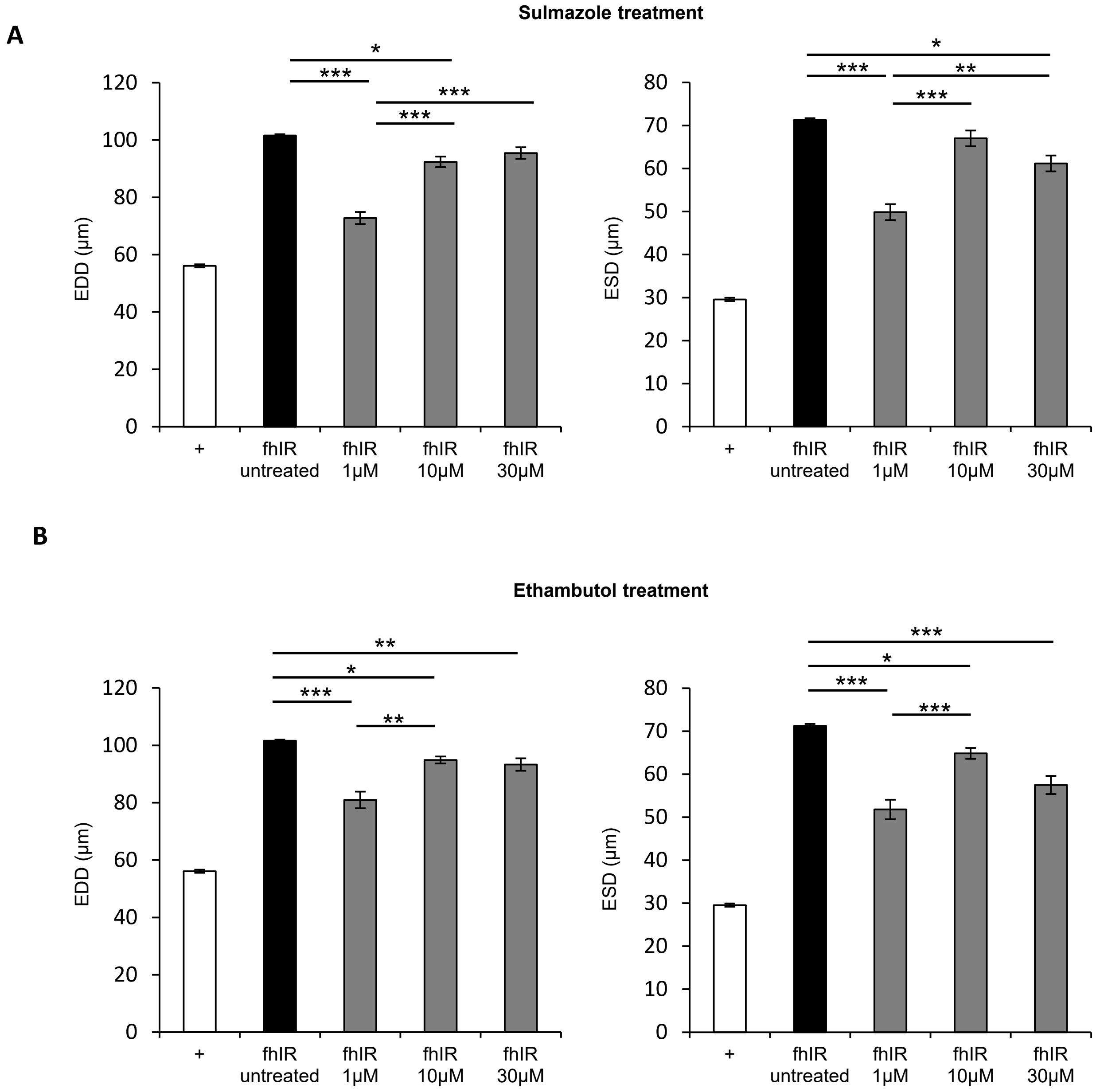

Figure S2 : Dose-dependent effects of sulmazole and ethambutol treatments.
End-Systolic Diameter (ESD, µm) and End-Diastolic Diameter (EDD, µm) of 4 to 6 days old *UAS-mitoGFP/+; Hand-GS/+*(+) control (n=569), and fhIR flies, untreated (n=1502) or treated with 1 µM (n=48), 10 µM (n=141) or 30 µM (n=51) sulmazole (Panel A) or with 1 µM (n=39), 10 µM (n=165) or 30 µM (n=57) ethambutol dihydrochloride (Panel B). All flies were fed with RU486 during both development (40 ng/ml of food) and adulthood (100 µg/ml). All values are medians (±SEM). Statistical significance between fhIR untreated or treated with the drugs was assessed by non-parametric Wilcoxon analysis. * $p<5.10^{-2}$. ** $p<1.10^{-3}$. *** $p<1.10^{-4}$.

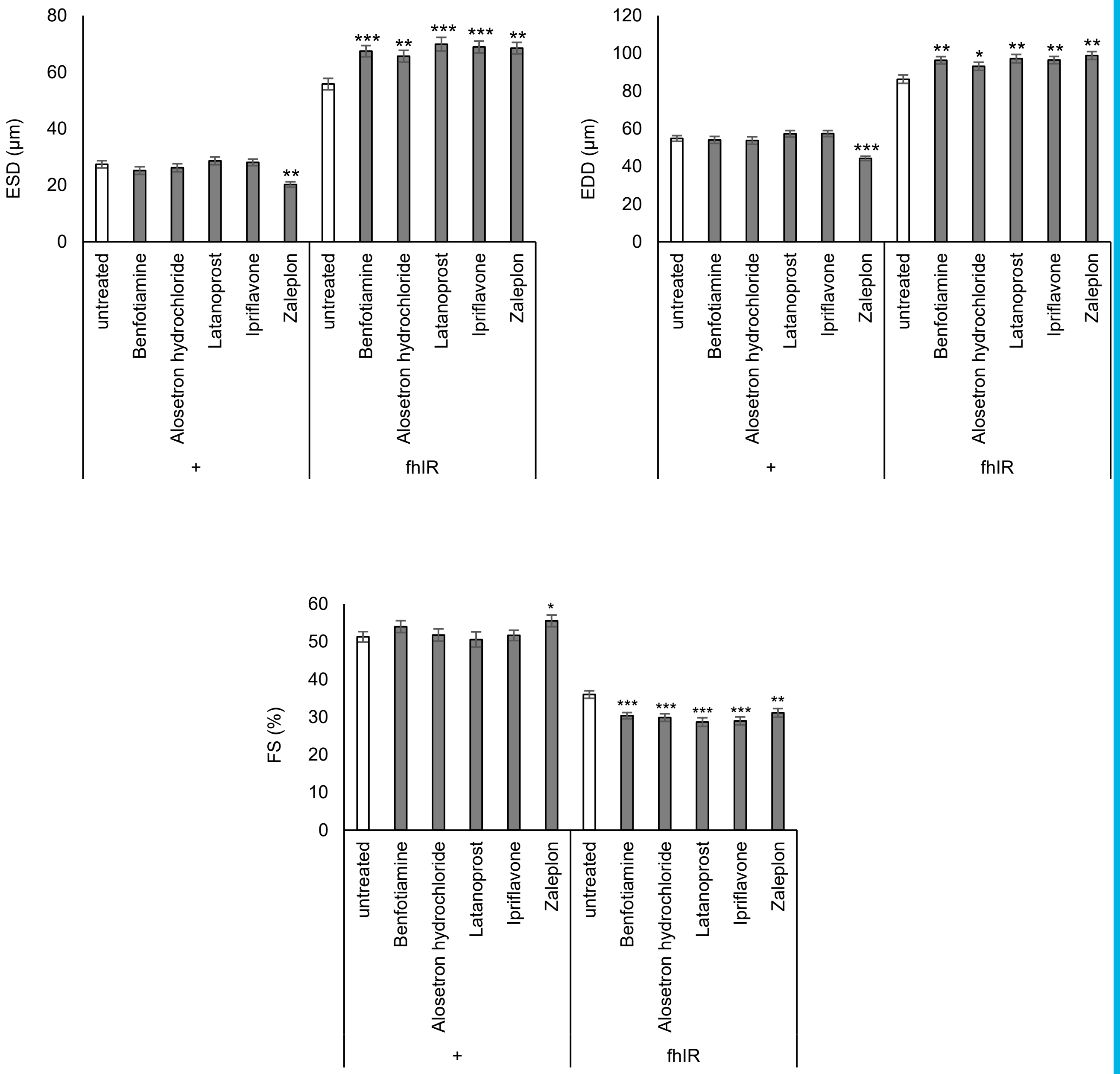

Figure S3 : Effects of the deleterious drugs identified in the screen on cardiac function of wild-type and frataxin-deficient flies.

A. End-Systolic Diameter (ESD, µm) of *UAS-mitoGFP/+; Hand-GS/+*(+) and *UAS-mitoGFP/UAS-fhIR; Hand-GS/+* (fhIR) flies, treated with DMSO (+: n=48, fhIR: n=64), with Benfotiamine (+: n=46, fhIR: n=52), Alosetron hydrochloride (+: n=46, fhIR: n=51), Latanoprost (+: n=35, fhIR: n=41), Ipriflavone (+: n=49, fhIR: n=52), or Zaleplon (+: n=38, fhIR: n=40).

B. C. End-Diastolic Diameter (EDD, µm) and Fractional Shortening (FS, %) of the same flies.

All flies were 5 days old, treated with drugs during development, and fed with RU486 during both development (40 ng/ml) and adulthood (100 µg/ml). All values are means (±SEM). Statistical significance was assessed by non-parametric Wilcoxon analysis. Significant differences between treated and untreated flies of the same genotype are indicated: * $p<5.10^{-2}$.** $p<1.10^{-3}$. *** $p<1.10^{-4}$.

Disease Models & Mechanisms • Supplementary information

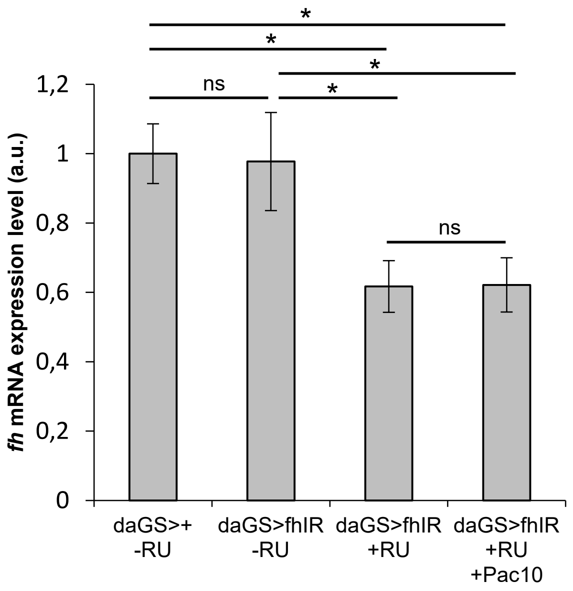

Figure S4: Paclitaxel treatment does not affect *fh* mRNA level.

qRTPCR analysis of mRNA extracts (five independent samples) from *daGS*>+ and *daGS>UAS-fhIR* third instar larvae, in which frataxin was downregulated by RNAi under the control of the ubiquitous and RU486-inducible *daGS* driver. Larvae were treated with 10 µM Paclitaxel or not, and treated with 2 µg/ml RU486 (+RU) or not (-RU), as indicated. *fh* mRNA level is given in arbitrary units (a.u). All values are means (±SEM). Statistical significance was assessed by non-parametric Wilcoxon analysis. Significant differences are indicated: * $p<5.10^{-2}$. ns: not specific.

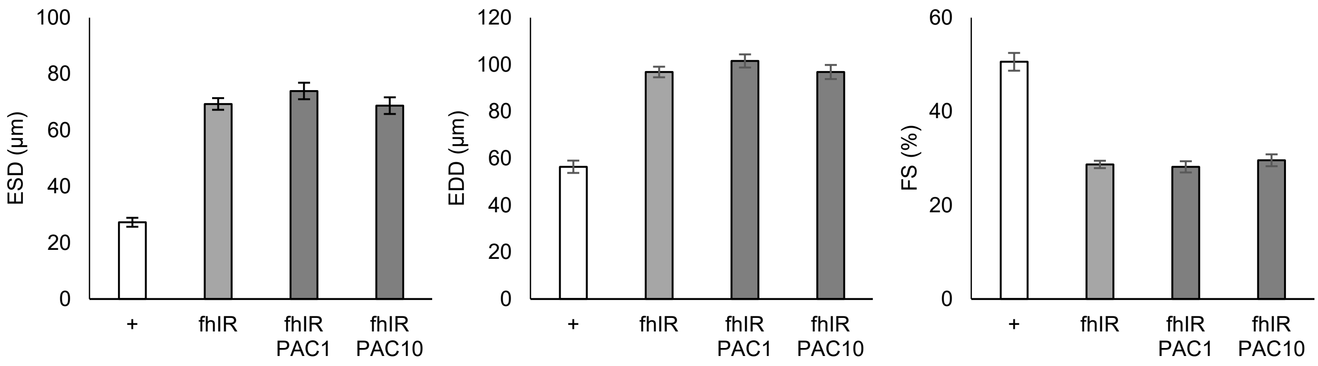

Figure S5: Post-symptomatic Paclitaxel treatment during adulthood does not improve heart function.

End-Systolic Diameter (ESD, µm), End-Diastolic Diameter (EDD, µm) and Fractional Shortening (FS, %) of 10 days old *UAS-mitoGFP/+; Hand-GS/+* (+) (n=28) and *UAS-mitoGFP/UAS-fhIR; Hand-GS/+* (fhIR) flies, treated during adulthood with DMSO (n=40), with 1µM Paclitaxel (n=35) or with 10µM Paclitaxel (n=33). All flies were fed with RU486 during both development (40 ng/ml of food) and adulthood (100 µg/ml). All values are means (±SEM). Statistical significance was assessed by non-parametric Wilcoxon analysis. No significant differences between fhIR flies treated during adulthood with Paclitaxel or untreated could be detected.

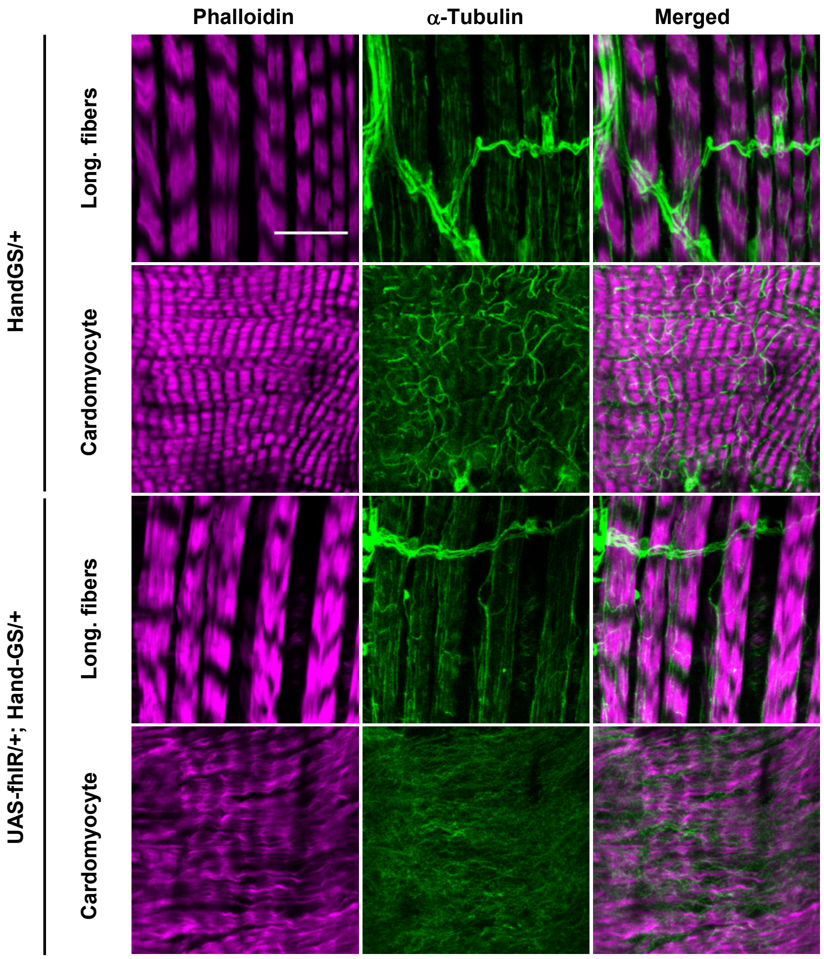

Figure S6: Actin and microtubule network in cardiomyocytes and heart longitudinal fibers of control and HandGS>fhIR flies .

Hearts of 3-5 days old *Hand-GS/+* and *UAS-fhIR/+; Hand-GS/+* adult male flies were dissected and double-labelled with phalloidin to stain F-actin and an anti-a tubulin antibody. For each genotype, sarcomeres of longitudinal fibers spreading along the ventral side of the heart (in which the HandGS driver is not expressed) and cardiomyocyte sarcomeres of the same fly are shown. Microtubule staining was also observed in nerve terminals connected to the longitudinal fibers. Flies were fed with RU486 during both development (40 ng/ml of food) and adulthood (100 µg/ml). Scale Bar: 10 µm.

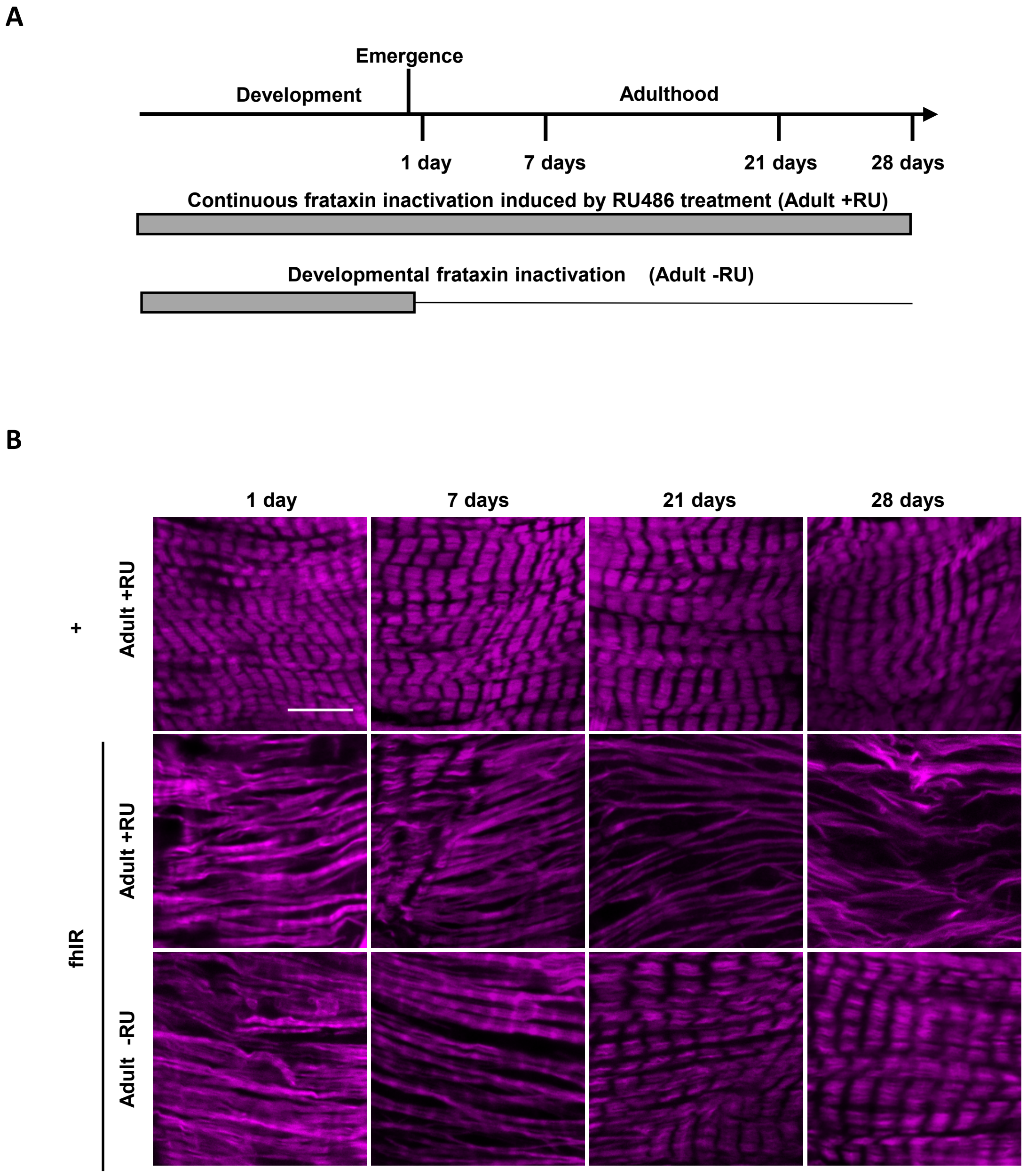

Figure S7: Reversibility of the sarcomeric defects in adult flies.
Scheme of the experiment. Adult +RU flies were treated with RU486 during development (1 µg/ml) and during adulthood (100 µg/ml). Adult –RU flies were treated with RU486 only during development. Heart dissections were performed 1 day, 7 days, 21 days and 28 days after adult emergence.
Organization of the actin network characterized with phalloidin staining in cardiomyocytes of control *UAS-mitoGFP/+; Hand-GS/+* (+) and *UAS-mitoGFP/UAS-fhIR; Hand-GS/+* (fhIR) adult males flies at various ages. In fhIR Adult-RU flies, four weeks after the arrest of RU486 treatment, a striated organization of the actin fibers was observed, when it was completely absent in fhIR flies continuously treated with RU486.

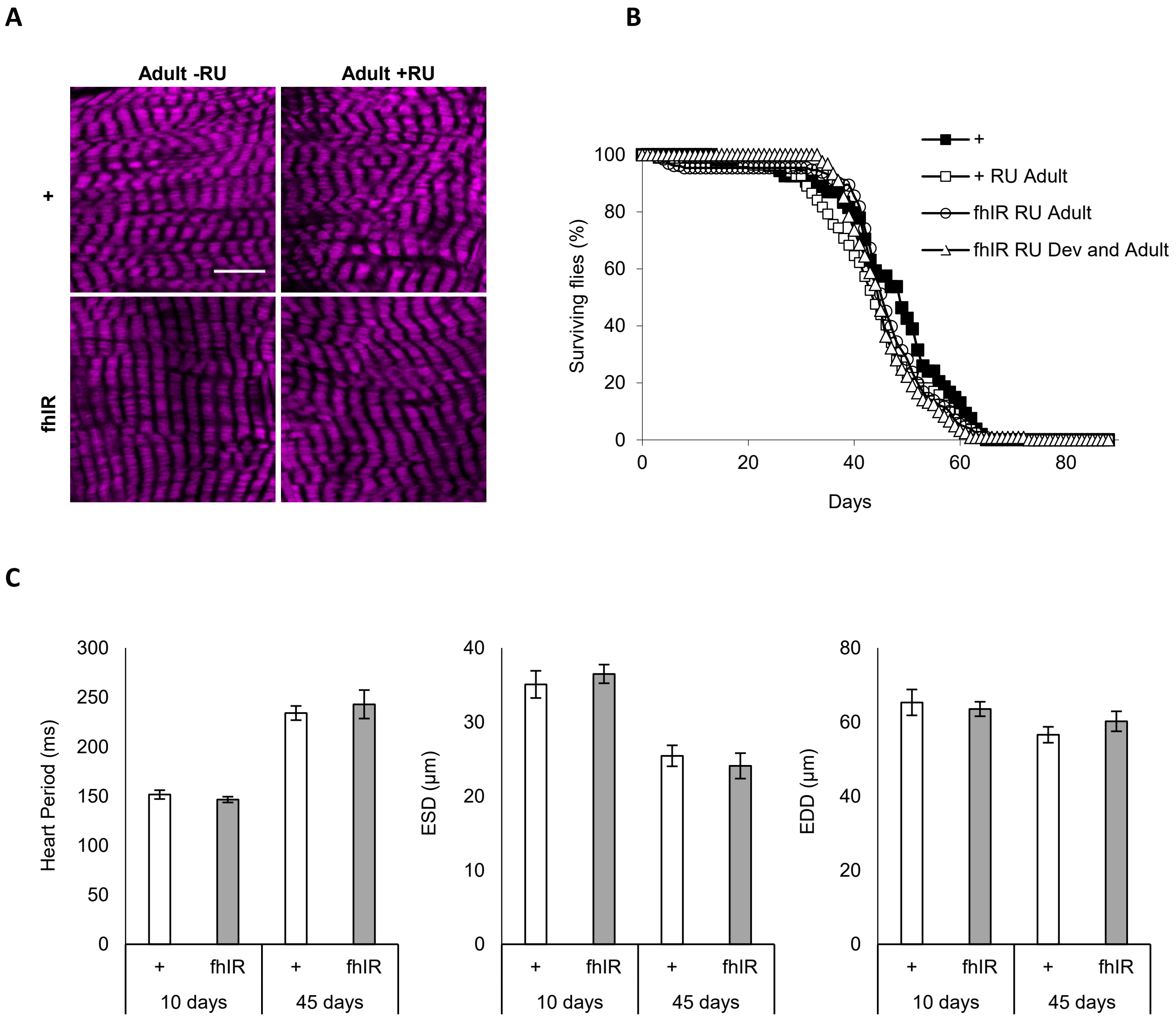

Figure S8: Absence of cardiac phenotypes following adult-specific frataxin inactivation.
Organization of the actin network characterized with phalloidin staining in cardiomyocytes of control *UAS-mitoGFP/+; Hand-GS/+* (+) and *UAS-mitoGFP/UAS-fhIR; Hand-GS/+* (fhIR) 10 days old adult males flies not treated with RU486 during development and treated with RU486 (100 µg/ml) during adulthood (Adult –RU) or not (Adult +RU).
Survival experiments on *UAS-mitoGFP/+; Hand-GS/+* male flies treated with RU486 during adulthood (+ RU Adult, n=82) or untreated (+, n=54) and *UAS-mitoGFP/UAS-fhIR; Hand-GS/+* treated with RU486 during adulthood only (fhIR RU Adult, n=131) or during development and adulthood (fhIR RU Dev + Adult, n=121).
Heart Period, ESD and EDD of *UAS-mitoGFP/+; Hand-GS/+* (+) and *UAS-mitoGFP/UAS-fhIR; Hand-GS/+* (fhIR) 10 days and 45 days old male flies treated with RU486 (100 µg/ml) only during adulthood. All values are means (±SEM). Statistical significance was assessed by non-parametric Wilcoxon analysis. No significant differences (ns) between fhIR and control flies of the same age could be detected.

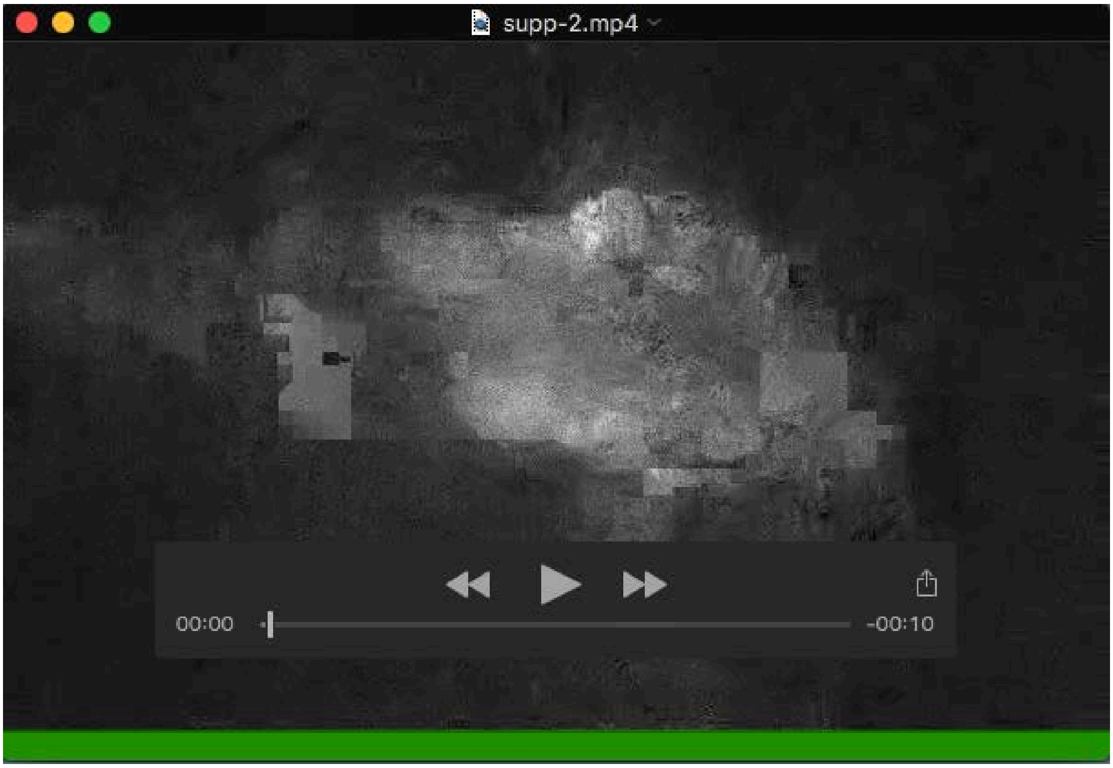

Movie 1: Heart beats of a control fly.

The Movie is acquired on a 4 days old *w/Y;UAS-mitoGFP/+; Hand-GS/+* male fly. The speed of the movie is 50 fps.

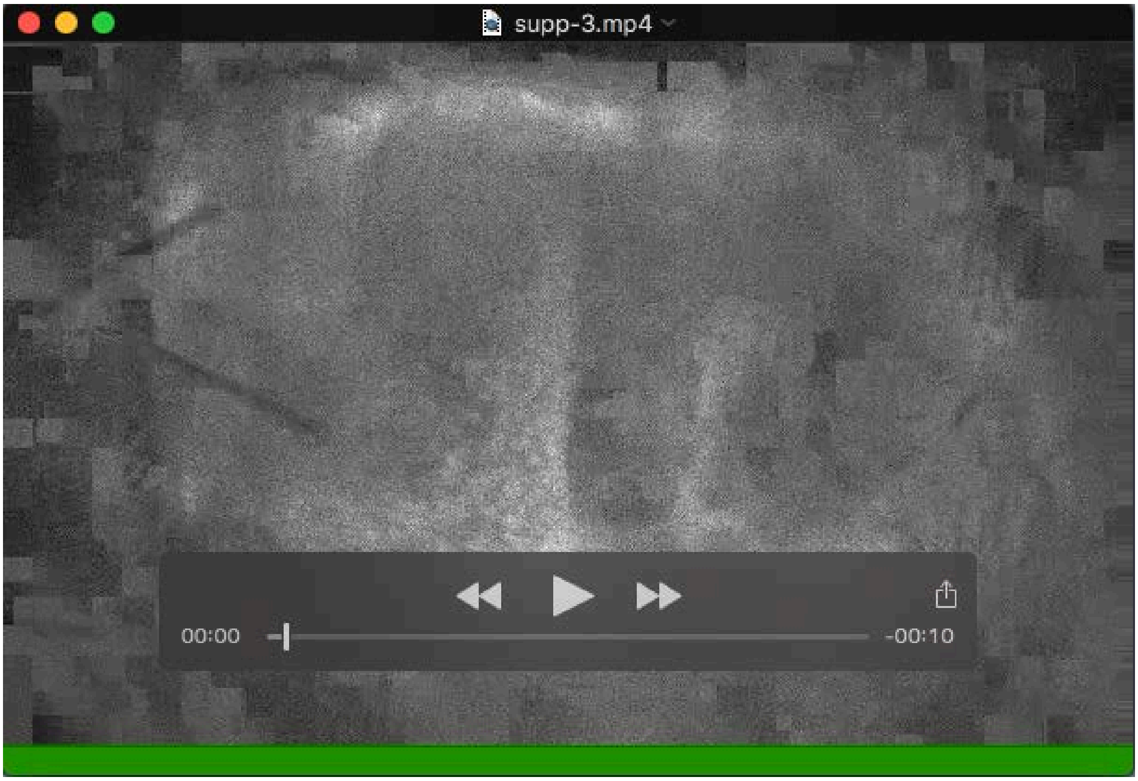

Movie 2: Heart beats of a frataxin deficient fly.
The Movie is acquired on a 4 days old *w/Y; UAS-mitoGFP/UAS-fhIR; HandGS/+* (fhIR) male fly. The speed of the movie is 50 fps.

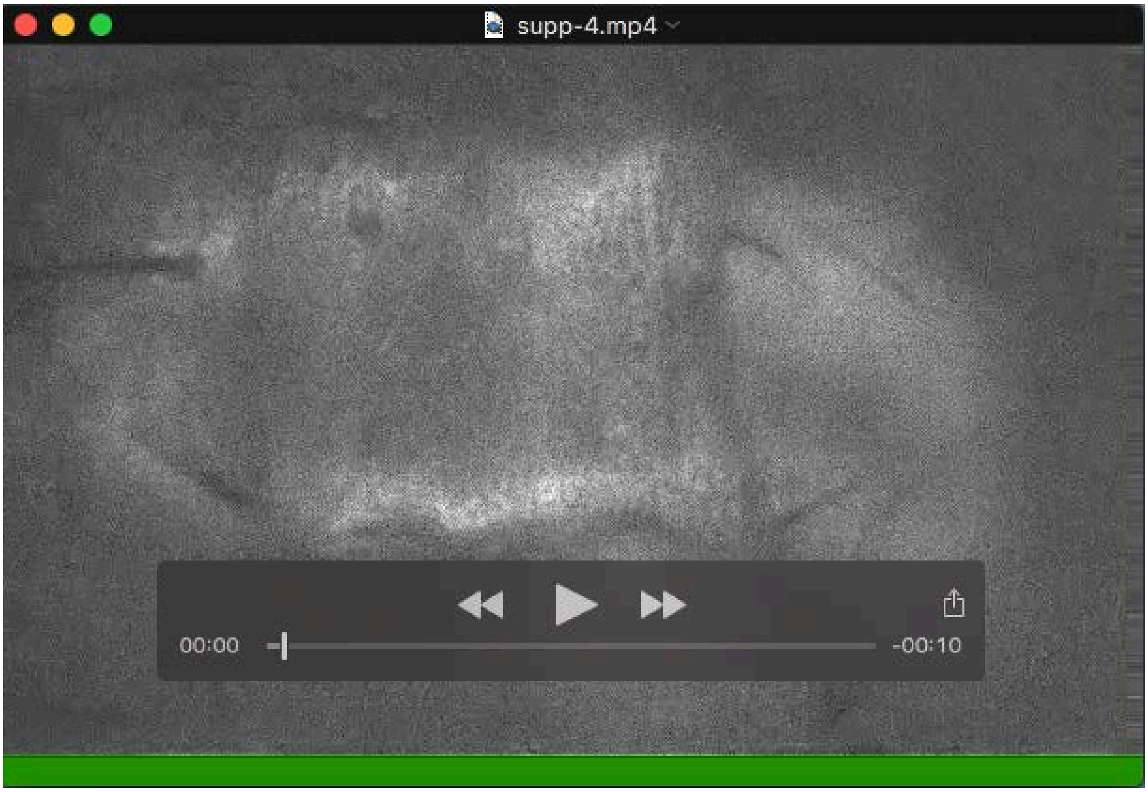

Movie 3: Heart beats of a frataxin deficient fly treated with Paclitaxel.
The Movie is acquired on a 4 days old *w/Y; UAS-mitoGFP/UAS-fhIR; HandGS/+* (fhIR) male fly, treated with 10µM of Paclitaxel during development and adulthood. The speed of the movie is 50 fps.
